# Estimating the return on investment of selected infection prevention and control interventions in healthcare settings for preparing against novel respiratory viruses: modelling the experience from SARS-CoV-2 among health workers

**DOI:** 10.1016/j.eclinm.2023.102388

**Published:** 2024-01-05

**Authors:** Ece Özçelik, Aliénor Lerouge, Michele Cecchini, Alessandro Cassini, Benedetta Allegranzi

**Affiliations:** a Organisation for Economic Co-operation and Development, 2 Rue André-Pascale, 75016, Paris, France; b Infection Prevention and Control Unit, Infectious Diseases Service, Lausanne University Hospital, Lausanne, Switzerland; c Health Emergencies Programme, World Health Organization, Avenue Appia, 1211 Geneva 27, Switzerland

**Keywords:** Pandemic preparedness, Infection prevention and control, IPC, Health professionals, COVID-19

## Abstract

**Background:**

Insufficient infection prevention and control (IPC) practices in healthcare settings increase the SARS-CoV-2 infection risk among health workers. This study aimed to examine the level of preparedness for future outbreaks.

**Methods:**

We modelled the experience from the COVID-19 pandemic and assessed the return on investment on a global scale of three IPC interventions to prevent SARS-CoV-2 infections among health workers: enhancing hand hygiene; increasing access to personal protective equipment (PPE); and combining PPE, with a scale-up of IPC training and education (PPE+). Our analysis covered seven geographic regions, representing a combination of World Health Organization (WHO) regions and the Organisation for Economic Co-operation and Development (OECD) countries. Across all regions, we focused on the first 180 days of the pandemic in 2020 between January 1st and June 30th. We used an extended version of a susceptible-infectious-recovered compartmental model to measure the level of IPC preparedness. Data were sourced from the WHO COVID-19 Detailed Surveillance Database.

**Findings:**

In all regions, the PPE + intervention would have averted the highest number of new SARS-CoV-2 infections compared to the other two interventions, ranging from 6562 (95% CI 4873–8779) to 38,170 (95% CI 33,853–41,901) new infections per 100,000 health workers in OECD countries and in the South-East Asia region, respectively. Countries in the South-East Asia region and non-OECD countries in the Western Pacific region were poised to achieve the highest level of savings by scaling up the PPE + intervention.

**Interpretation:**

Our results not only support efforts to make an economic case for continuing investments in IPC interventions to halt the COVID-19 pandemic and protect health workers, but could also contribute to efforts to improve preparedness for future outbreaks.

**Funding:**

This work was funded by 10.13039/100004423WHO, with support by the German 10.13039/501100003107Federal Ministry of Health for the 10.13039/100004423WHO10.13039/100006190Research and Development Blueprint for COVID-19.

**Research in context**

**Evidence before this study**

We searched PubMed, MEDLINE, Embase and MedRxiv since the beginning of the COVID-19 pandemic to August 14, 2023 for original research articles focusing on estimating the cost-effectiveness of selected IPC interventions across several countries, i.e., enhancing hand hygiene, increasing access to personal protective equipment, and increasing access to personal protective equipment (PPE) in combination with IPC training and education (PPE+). We used the search terms: ‘COVID-19’; ‘SARS-CoV-2’; ‘hand hygiene’; ‘personal protective equipment’; ‘PPE’; ‘mask’; ‘glove’; ‘gown’; ‘training’; ‘education’; ‘infection prevention’; ‘infection control’; ‘IPC’; ‘infection prevention and control’; ‘economic evaluation’; ‘cost-effectiveness'; ‘health worker’; ‘healthcare worker’; ‘healthcare professional’; ‘health professional’; ‘physician’; ‘nurse’. Our search showed that only one study assessed the cost effectiveness of scaling-up PPE for health workers in low- and middle-income countries (LMICs). The authors reported that an investment of US$ 9.6 billion was required to protect health professionals in all LMICs, equivalent of an almost an 8% return on investment.

**Added value of this study**

This is the first study that generated cost-effectiveness estimates for three IPC interventions recommended by WHO to reduce the burden of SARS-CoV-2 infections among health workers in seven geographic regions worldwide.

We found that improved access to PPE for 80% of health workers could have averted new SARS-CoV-2 infections across all regions. The effectiveness of this intervention would have been substantially enhanced by combining it with increased access to IPC education and training. In many regions, hospitalisations, intensive care unit (ICU) admissions and deaths among health workers could also have been averted by scaling-up PPE only and PPE + interventions. The African, South-East Asia and Eastern Mediterranean regions, and non-OECD countries in Latin America and Europe were poised to achieve the highest health and economic gains by investing in IPC measures.

**Implications of all the available evidence**

Deliberate investments in bolstering IPC capacity and programmes can not only halt the COVID-19 pandemic and protect health workers, but also contribute to efforts to improve preparedness for future outbreaks across all regions.

## Introduction

The COVID-19 pandemic exacted a staggering toll on health workers across the globe. Since the outset, health workers faced an elevated risk of SARS-CoV-2 infection and premature loss of life.^1^^,^^2^ The burden of COVID-19 among health workers also hampered the ability of health systems to respond to the pandemic.

Strengthening infection prevention and control (IPC) measures in healthcare settings reduces the risk of healthcare-associated infections (HAIs), including SARS-CoV-2 infections.^2^^,^^3^ However, global IPC capacity was already marked by striking gaps before the pandemic,^4^, ^5^, ^6^, ^7^ despite the availability of clear recommendations and implementation strategies for effective IPC programmes.^5^^,^^8^^,^^9^ Recently, the World Health Organization (WHO) issued the first global report on IPC, which highlighted significant cross-country differences in the implementation levels of IPC programmes.^10^

Demonstrating the cost-effectiveness of IPC interventions and return on investment are critical elements to secure buy-in from policymakers who make decisions related to resource allocation for health. Previous studies showed that IPC interventions offer a cost-effective strategy for reducing infections in healthcare settings,^11^ although relatively little is known about the cost-effectiveness of IPC interventions in the context of the COVID-19 pandemic.^12^ However, previous studies also emphasised that available evidence suffers from important methodological weaknesses.^11^ Furthermore, they highlighted that the nuances in the ways countries design and implement IPC interventions in their own settings make it difficult to compare the effectiveness of IPC interventions across countries and regions over time.^11^

This study aims to examine the level of preparedness for future outbreaks, particularly those that can be caused by novel respiratory viruses. Specifically, we model the experience from the COVID-19 pandemic and assess the return on investment of three selected IPC interventions to prevent SARS-CoV-2 infections among health workers in the first six months of the COVID-19 pandemic: enhancing hand-hygiene; increasing access to personal protective equipment (PPE) for 80% of health workers (‘PPE only’); and combining increased access to PPE with scale-up of IPC training and education (‘PPE+’). We selected these three ICP interventions as 1) they are part of the WHO core components and minimum requirements of IPC programmes,^8^^,^^9^ 2) they are relevant to reduce HAIs in general, and SARS-CoV-2 infections in particular^13^ and 3) previous studies suggested that they offered an effective strategy to interrupt the spread of SARS-CoV-2 infections.^2^^,^^3^^,^^14^

## Methods

### Study design

Our analysis covers seven geographic regions worldwide, representing a combination of WHO regions and the 38 countries in the Organisation for Economic Co-operation and Development (OECD) (Appendix pp. 1–4). Across all regions, the analysis includes the first 180 days of the pandemic in 2020 between January 1st and June 30th.

During the COVID-19 pandemic, mathematical models were frequently used for various purposes.^15^ Broadly, COVID-19 models were grouped into two categories: forecasting models and mechanistic models, though hybrid approaches were also deployed.^16^ While forecasting models can be a valuable tool to generate short-term projections to guide policy decisions, they do not account for the underlying dynamics of disease transmission and they are less suitable in the ex-post assessment of alternative policy scenarios. Mechanistic models (e.g., the basic susceptible-infectious-recovered [SIR] framework) replicate the dynamics of SARS-CoV-2 by incorporating the key disease characteristics, though these models are limited by the availability of evidence on the epidemiological features of a pathogen.^16^

### Ethics

The study did not require ethical approval. Our study uses data from publicly available datasets, as well as the WHO COVID-19 Detailed Surveillance Database and the 2019 WHO IPC Self-Assessment Framework. Data extracted from the WHO COVID-19 Detailed Surveillance Database are aggregated without any personal identifiers, which were notified to WHO through the surveillance system developed specifically for COVID-19. The 2019 WHO IPC Self-Assessment Framework is a published survey of IPC implementation in health facilities and information stemming from the scientific literature. Therefore, informed consent was not required.

### Statistical analysis

**Fig. 1:**
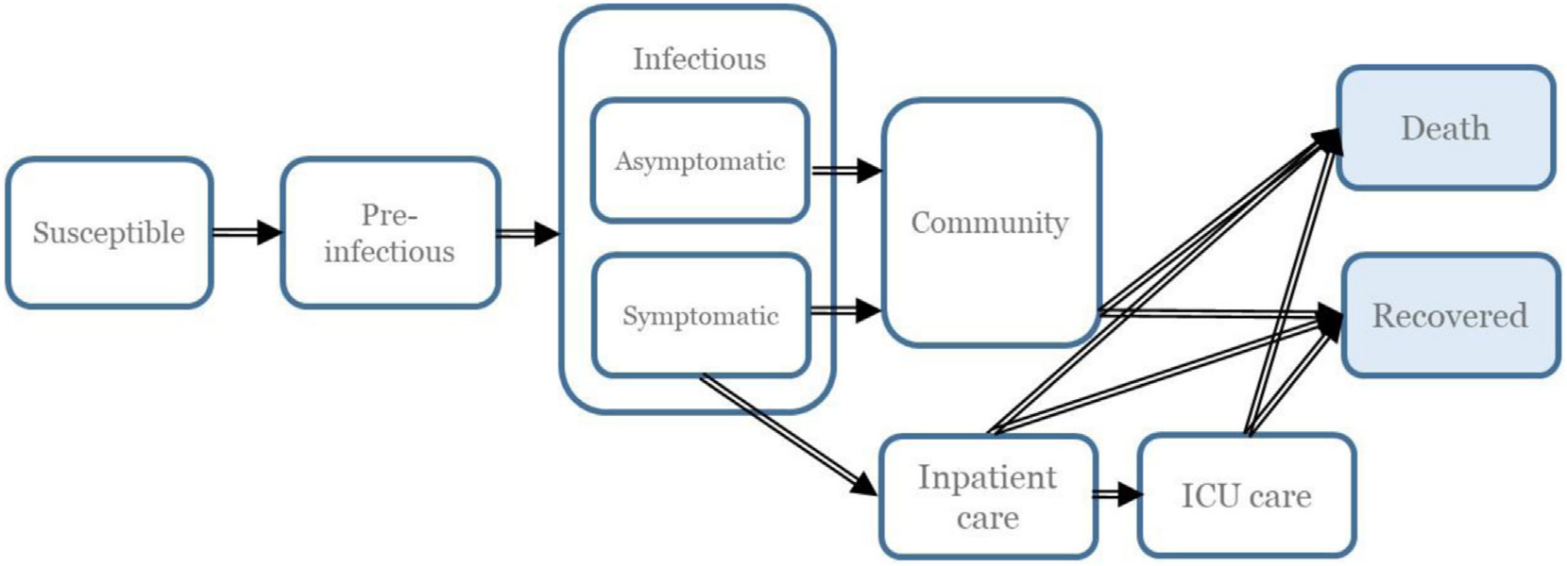
**Structure of the OECD SPHeP-COVID-19 model**. Arrows represent the flow of individuals. Transition states are denoted by white boxes and absorbing states are denoted in light blue boxes.

In our study, we used the OECD Strategic Public Health (SPHeP)-COVID-19 model to simulate the disease dynamics of SARS-CoV-2 in the first 6 months of the pandemic. It is a mechanistic model that extends the basic SIR framework. The OECD model was developed as a region-specific, age-stratified compartmental model that uses discrete time steps of one day. It categorises individuals in a closed population into mutually exclusive groups based on disease status, i.e., susceptible, pre-infectious, infectious, and recovered (Fig. 1). The model distinguishes between health workers and community members in terms of the relative risk of SARS-CoV-2 infections in order to consider both the higher risk faced by health workers and the broader risk arising from the epidemiology in the community.

At the outset, the simulation assumed that all individuals were susceptible, except those considered seeding. As individuals advanced through compartments, they were first infected, but could not spread the virus (*pre-infectious*). Next, they became infectious and could be admitted to inpatient care and the ICU, depending on their symptomatology and the availability of hospital/ICU beds. (Appendix pp. 5 and 6). Finally, infected individuals could end up in one of the two absorbing states, i.e., recovery or death due to COVID-19.

Table 1 presents the key model parameters.^17^, ^18^, ^19^, ^20^ The model consists of four modules. The first module allowed to track the demographic dynamics of a given population, such as births and deaths, as extracted from the United Nations Population Division for the general population for 2019.^22^ The WHO National Health Workforce Accounts Portal was used to gather data on the country-specific number and age distribution of health workers.^22^^,^^23^ Professional categories included physicians, nurses, midwives, dentists and pharmacists. Second, the SARS-CoV-2 exposure module was designed to replicate the epidemiology of the original strain of the virus. We used country-aggregated data on the number of SARS-CoV-2 infections among health workers and in the general public from the WHO COVID-19 Detailed Surveillance Database (Appendix pp. 7–15). This module also considered the physical distancing measures in the community. The third module replicated the physical resources available in each region, which were measured using an updated version of hospital and ICU bed capacity estimates generated by Walker and colleagues based on data derived from the literature and the World Bank.^21^ Finally, the fourth module yielded the output on the health and economic impact of the pandemic produced by the interaction of the previous modules.

**Table 1:** Key model parameters used in the OECD SPHeP COVID-19 model.

Parameter	Values used in the model	Reference	Parameter varies by age
**Epidemiology of SARS-CoV-2**			
Incubation period (days)	μ = 5.1	17	No
Infectivity rate	Infectivity starts 2 days prior to the onset of symptoms, followed by an exponential decrease to match the infectious period.	Working assumption	No
Infectious period (days)	μ = 9	18	No
Basic reproduction number ($R_{0})$	Baseline $R_{0}$ presented in the main analysis = 2.5 Varying scenarios from $R_{0}=1.5$ through $R_{0}=5$ (inclusive)		No
Share of asymptomatic patients	Children (0–18 years): 46.7% (95% CI 32.0–62.0) Adults (19–59 years): 32.1% (95% CI 22.2–43.9) Elderly (≥60 years):19.7 (95% CI 12.7–29.4)	19	Yes
Infectiousness of asymptomatic individuals relative to symptomatic patients	50%	Working assumption based on findings from.^20^	No
**Access to healthcare services**			
Likelihood of access to hospital care	Access to hospital care is assumed to be the same for all individuals aged 75 years and less. Between the ages of 75–85 years, access to care is assumed to decline linearly. For individuals over 85 years, 66% of individuals are assumed to have access to care.	Working assumption	Yes
	Access to hospital care depends on hospital bed occupancy. We assume that all people have equal access to hospital care when hospital bed occupancy is below 50%. When hospital bed occupancy is over 50%, a linear decline in access to hospital care is assumed, reaching no access when all hospital beds are occupied.	Working assumption	No
	Health workers are assumed to have uninterrupted access to healthcare services until hospital bed capacity reaches full capacity.	Working assumption	No
Likelihood of access to ICU care	Access to ICU care depends on ICU bed occupancy. When ICU bed occupancy is below 50%, all individuals in the simulation are assumed to have equal access to ICU care. When ICU bed occupancy is over 50%, access to ICU care is assumed to decline linearly. It is assumed that no individual has access to ICU care when 150% of ICU beds are occupied.	Working assumption	No
**Utilisation of healthcare services**			
Mean duration of symptomatic cases in the community (non-hospitalised)	10 days	Refers to self-isolation period for health workers as per WHO guidelines	No
From onset to admission to hospital	4 days	Values in the literature range from 1.2 to 12 days.	No
Mean duration of hospitalisation for non-critical cases if survival	9 days	21	No
Mean duration of hospitalisation for non-critical cases if death	9 days	21	No
Mean duration of ICU care if survival	14.8 days	21	No
Mean duration of ICU care if death	11.1 days	21	No
Mean duration of stepdown following ICU admission	3 days	21	No
Mean duration of hospitalisation if ICU care required, but not received, followed by death	1 day	21	No
Mean duration of hospitalisation if ICU care required, but not received, followed by survival	7.4 days	21	No
Probability of death if ICU care required, but not received	95%	21	No
Probability of death if hospitalisation required, but not received	60%	21	No

The model builds on several assumptions. First, it is assumed that the epidemiology of SARS-CoV-2 remained constant over the analysis period. Second, we assumed that recovered individuals acquired immunity and could not be re-infected in the remainder of the simulation. Third, it was assumed that no major developments occurred in the availability of therapeutics to treat SARS-CoV-2 infections that could significantly alter the course of hospital stay during the simulation. We did not make any explicit assumptions about the transmission patterns of SARS-CoV-2 (e.g., between health workers or between health workers and the general community). We also did not explicitly model how the changes in the early screening, detection and surveillance for COVID-19 in healthcare settings might have influenced the spread of SARS-CoV-2 infections or the effectiveness of the IPC interventions. Finally, the initial SARS-CoV-19 infections were assumed to be imported from outside the national borders, with one case per 100 000 inhabitants assumed to be imported in the first 30 days of the pandemic. This assumption implied that no new cases were transmitted between countries via cross-border travel, except during the initial days of the pandemic. We opted for this assumption to reflect the freeze on international travel instituted in many countries in the initial months of the pandemic.

Similar to Risko and colleagues (2020), we used an auto-stop trigger to take into account the different social distancing measures that were scaled up during the COVID-19 pandemic.^12^ The auto-stop was triggered when the daily death rate reached 1.6 per 100 000 population, and mitigation measures were assumed to be put in place to curb transmission. It was assumed that 7 days after the auto-start is triggered, a linear decline toward 0.8 would be observed in the reproduction number. When the suppression target of 0.8 deaths per 100,000 population was reached, the reproduction number was reset to one-half of the initial $R_{0}$, assuming that some restrictions would remain in place.

### Model output

Health impacts captured morbidity and mortality attributable to COVID-19 and included the number of averted infections, averted admissions to inpatient/ICU care and deaths, and disability-adjusted life years (DALYs) averted. The economic impact quantified medical costs incurred due to treating SARS-CoV-2 infections and productivity loss among health workers owing to absence from work (Appendix pp. 15–23).

### Modelling interventions

To gauge their population-level effectiveness, selected interventions were evaluated against a *business-as-usual* scenario, assuming that no new IPC interventions were rolled out in the course of the simulation. The comparison between the *business-as-usual* and intervention scenarios corresponded to the impact of an intervention. Interventions were modelled separately.

As shown in Table 2, the design features of the modelled interventions reflected WHO guidelines and were not necessarily those in place in each country (Appendix pp. 24–38).^2^^,^^14^^,^^24^ Each intervention was modelled across four key parameters: 1) effectiveness of the intervention at the individual level; 2) time to maximum effectiveness achieved and effectiveness over time; 3) intervention coverage; and (4) implementation costs. We determined the individual-level effectiveness estimates in several steps. First, we critically reviewed systematic reviews and meta-analyses relevant to ongoing and past outbreaks, whenever possible (Appendix pp. 24–39). Next, we discussed our selection of estimates based on the availability and quality of evidence from the literature with a group of IPC experts convened by WHO. We used facility-level data collected from 81 countries through the 2019 WHO IPC Self-Assessment Framework to determine the level of coverage at baseline for each modelled intervention for each region (Appendix pp. 39 and 40).^7^ This step allowed us to estimate the share of health facilities in each region that had IPC capacity in line with the WHO IPC core components at the beginning of our analysis period.^8^ Consistent with WHO-CHOICE guidelines, the target coverage for each intervention was set at 80%.^25^ We calculated time to the maximum effectiveness for each intervention as a steady state, such that each intervention was assumed to be put in place prior to the beginning of the outbreak. We assumed that the effectiveness of these interventions remained unchanged over the study period. The provision of preventive and health services was assumed to remain unchanged. The analysis of per capita costs followed the WHO-CHOICE Framework.^25^ Costs were calculated as a combination of programme-level costs, including administration, training and other activities, and health worker-level expenditure, such as purchasing PPE (Appendix pp. 41–45). Costs were assumed to be borne by governments as an upfront cost. Per capita costs were expressed in 2020 US$ at purchasing power parity. They were additional to those that existed before the COVID-19 pandemic and included expenditure by the healthcare sector.

**Table 2:** Overview of key design features of the modelled interventions.

	Enhance hand hygiene	Increased access to PPE (PPE only)	Combine increased access to PPE and IPC training and education (PPE+)
Intervention design	Bundle intervention consisting of 5 components: • Procuring cleaning facilities/equipment • Hand hygiene training and education (2-h training at health facility by IPC focal point with dedicated time per 250 beds) • Hand hygiene guidelines and visual reminders • Audit and feedback • Creating a work environment that promotes best practices in hand hygiene	PPE programme with 4 components: • PPE tracking system • Procuring PPE in line with WHO guidelines	IPC training and education programme with 3 components: • IPC training and education (2-h training at health facility by IPC focal point with dedicated time per 250 beds) • IPC guidelines and visual reminders • Feedback mechanism *This intervention builds on the assumption that health workers already have access to PPE at target coverage levels.*
Estimated effect size	Risk of infection: enhanced hand hygiene for adults vs. none: relative risk, 0.85; 95% CI 0.79–0.92 based on reference.^24^	Risk of infection: access to and using PPE vs. none: relative risk, 0.36; 95% CI 0.24–0.53 (authors' calculations based on reference.^14^	Risk of infection: IPC training vs. none: relative risk, 0.11; 95% CI 0.08–0.15 (authors' calculations based on references^2^ and.^14^
Intervention coverage at baseline	Varies by region: 7%–56%	Varies by region: 10%–51%	Varies by region: 4%–37%
Target coverage	80%	80%	80%
Time to the maximum effectiveness and effectiveness over time	It is assumed that the intervention is scaled-up to target coverage before the start of the simulation and effectiveness remains at the same level over the study period.	It is assumed that the intervention is scaled-up to target coverage before the start of the simulation and effectiveness remains at the same level over the study period.	It is assumed that the intervention is scaled-up to target coverage before the start of the simulation and effectiveness remains at the same level over the study period.
Per capita intervention cost	Varies by region: US$ 0.14–US$ 0.52	Varies by region: US$ 0.34–US$ 1.24	Varies by region: US$ 0.47–US$ 2.80

Note: Values for intervention coverage at baseline and per capita intervention costs by region are provided in the Supplementary materials.

### Propagating uncertainty and model validation

Simulation uncertainty was calculated by running each scenario, including both the *business-as-usual* and intervention scenarios, independently and randomly 20 times, which yielded 20 independently drawn subsamples. Uncertainty on the effectiveness of each intervention was derived by running each intervention scenario nine times, each time using an effectiveness value that was set to be uniformly distributed between the minimum, middle and maximum values of the 95% confidence intervals (CIs) of the relative risk derived from the systematic reviews and meta-analysis. The mid-effectiveness of each intervention and its 95% CIs, which account for both the simulation and intervention uncertainty, were then estimated by calculating the difference between the $i^{th}$ subsample of the *business-as usual* scenario with the $i^{th}$ subsample in the intervention scenario, where *i* was between 1 and 20, corresponding to the number of times the model was run for each scenario to incorporate the simulation uncertainty. The same process was performed nine times—one for each draw of the intervention effectiveness—each time maintaining the same value for the $i^{th}$ subsample in the *business-as-usual* scenario. In the final step, the mid-effectiveness of the intervention and its 95% CIs were estimated on the 180 non-independent subsamples by using the quantile estimation from a sample methodology.

Economic outputs were validated against previous estimates generated by Tan-Torres and colleagues (2020).^4^ We also carried out several sensitivity checks and additional analyses (Appendix pp. 45–57). First, we examined the extent to which our estimates were sensitive to different $R_{0}$ scenarios. Second, we examined the differences in the effectiveness of the modelled interventions under an unmitigated pandemic scenario. Third, we investigated whether our estimates to discern the effectiveness of PPE only and PPE + interventions were robust to using different methodologies. Fourth, we re-run our principal analysis, but this time using pro-rated costs. Finally, as an additional analysis, we assessed a scenario under which each modelled intervention was implemented across all healthcare settings (Appendix pp. 57–60). All analyses were conducted using Python.

### Role of the funding source

Authors from the WHO funding body were involved in the study design, data collection, data interpretation, and writing of the report. All authors were involved in data interpretation, reviewed drafts of the manuscript, and provided critical input. All authors approved the final version of the manuscript.

## Results

Across all regions, increasing access to PPE for 80% of health workers prior to the pandemic would have averted SARS-CoV-2 infections among health workers, with the new infections averted per 100,000 health workers ranging from 2291 (95%CI 667–3649) in OECD countries to 20,983 (95% CI 9218–30,551) in the South-East Asia region. The effectiveness of this intervention could have been substantially enhanced in all regions if it had been combined with IPC training and education (PPE+). The hand hygiene intervention could have produced more modest health gains. In addition, the PPE only and PPE + interventions could have also averted hospitalisations, ICU admissions and deaths, although the magnitude of the estimated gains were smaller in comparison to infections averted. The potential protective effects of the hand hygiene intervention in terms of preventing hospitalisations, ICU admissions and deaths did not reach statistical significance for any of the regions included in the analysis.

**Fig. 2:**
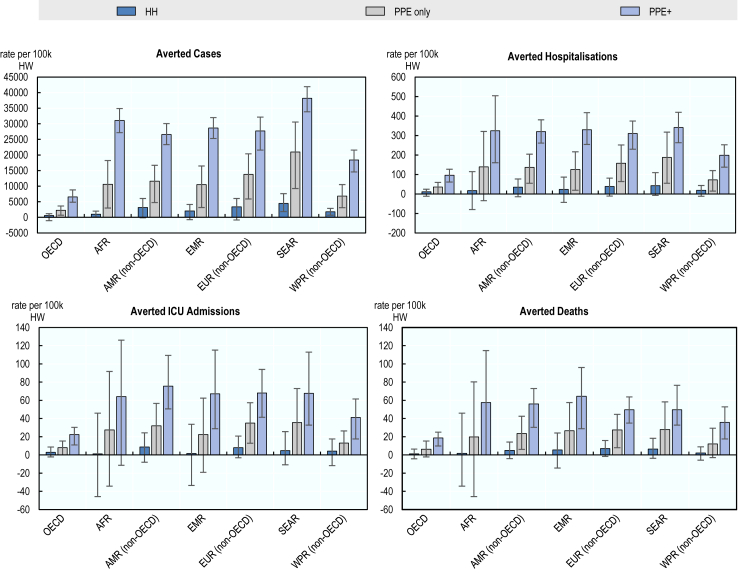
**Averted cases, hospitalisations, ICU admissions and deaths attributable to the modelled IPC interventions per 100,000 health workers, by regions**. Dark blue represents enhancing hand hygiene (HH); grey represents increasing access to PPE (PPE only); purple represents increasing access to PPE in combination with IPC training and education (PPE+). Error bars represent the 95% confidence intervals for each region.

As shown in Fig. 2, the PPE + intervention would have averted an estimated 38,170 (95% CI 33,853–41,901) new infections per 100,000 health workers in the South-East Asia region. This was followed by the African region and non-OECD countries in Europe where the PPE + intervention would have averted 31,052 (95% CI 27,174–34,890) and 27,716 (95% CI 21,583–32,156) new infections per 100,000 health workers, respectively. The lowest level of gains would have been realised in OECD countries where scaling-up the PPE + intervention could have averted an estimated 6562 (95% CI 4873–8779) new infections per 100,000 health workers. The magnitude of hospitalisations averted through the PPE + intervention would have been similar across countries in the African, the Eastern Mediterranean and South-East Asia regions, as well as in non-OECD countries in the Americas and European regions, ranging from 341 (95% CI 262–419) to 310 (95% CI 229–374) per 100,000 health workers. Among non-OECD countries, the number of ICU admissions that could have been averted ranged from 41 (95% CI 18–62) in the Western Pacific region to 76 (95% CI 51–109) per 100,000 health workers in the Americas. In comparison, the number of deaths that could have been avoided ranged from 36 (95% CI 18–53) deaths per 100,000 health workers in the non-OECD countries in the Western Pacific region to 65 (95% CI 29–96) in the Eastern Mediterranean region. Compared to other regions, the protective effects of the PPE + intervention were estimated to be substantially lower in OECD countries across all health outcomes studied.

**Fig. 3:**
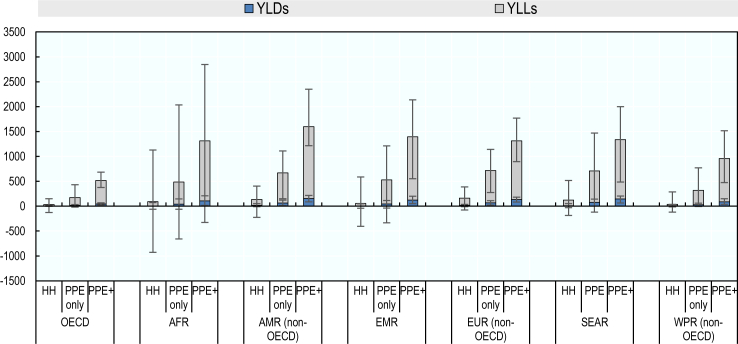
**Years lived with disability and years of life lost averted attributable to the modelled IPC interventions per 100,000 health workers**. Modelled IPC interventions are enhancing hand hygiene (HH), increasing access to PPE (PPE only) and increasing access to PPE in combination with IPC training and education (PPE+). Dark blue represents years lived with disability; grey represents years of life lost. Error bars represent the 95% confidence intervals for each region.

The PPE only and PPE + interventions would have generated the greatest health gains across all regions (Fig. 3). The PPE + intervention was associated with the greatest gains in life years, with years of life lost prevented ranging from 472 (95% CI 333–639) in OECD countries to 1443 (95% CI 1064–2198) per 100,000 health workers in the non-OECD countries in the Americas region. In most regions, the PPE + strategy was estimated to avert DALYs more than two-fold compared to the PPE only strategy. The PPE + strategy could have generated the greatest gains in DALYs averted in non-OECD countries in the Americas, followed by the Eastern Mediterranean, South-East Asia and African regions.

**Fig. 4:**
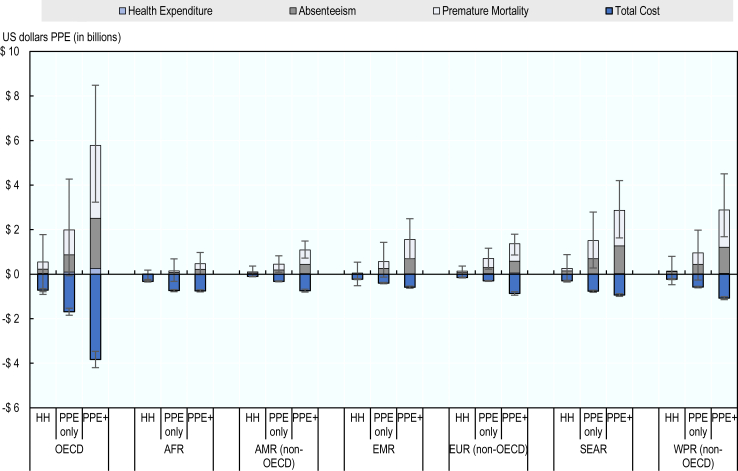
**Costs and savings associated with scaling-up the modelled IPC interventions, by regions**. Modelled IPC interventions are enhancing hand hygiene (HH), increasing access to PPE (PPE only) and increasing access to PPE in combination with IPC training and education (PPE+). Purple represents health expenditure; grey represents absenteeism; light blue represents premature mortality and dark blue represents total cost. Error bars represent the 95% confidence intervals showing the uncertainty associated with total savings and total costs. Savings are expressed in positive US dollars values and costs are expressed in negative US dollar values.

The PPE + intervention could have resulted in the highest level of net savings across all regions (Fig. 4). By contrast, the hand hygiene intervention was associated with the lowest level of estimated savings. Net savings associated with the PPE + intervention were highest in South-East Asia and non-OECD countries in the Western Pacific region, with estimated net savings of approximately US$ 1.94 billion (95% CI 0.75–3.2) and US$ 1.81 billion (95% CI 0.68–3.4), respectively. In all regions, savings were driven primarily by averting premature mortality, followed by absence from work and attributable healthcare expenditure.

The average estimated cost of the hand hygiene intervention was US$ 0.27 (95% CI 0.24–0.29) per capita adjusted for the differences in purchasing power across the seven regions, whereas costs of the PPE only and PPE + interventions were US$ 0.63 (95% CI 0.58–0.68) and US$ 1.15 (95% CI 1.05–1.25) per capita, respectively. The estimated per capita cost of the hand hygiene intervention was lowest in non-OECD countries in the Western Pacific region (US$ 0.14 [(95% CI 0.12–0.19]). The estimated per capita costs were nearly four times higher in OECD countries (US$ 0.52 [(95% CI 0.47–0.58]) and more than three times higher in non-OECD European countries. Among all seven regions, South-East Asia had the lowest estimated per capita cost of scaling-up the PPE + intervention (US$ 0.47 [(95% CI 0.42–0.60]). However, estimated costs were on average around six times higher in OECD countries and more than five times higher in non-OECD European countries.

**Fig. 5:**
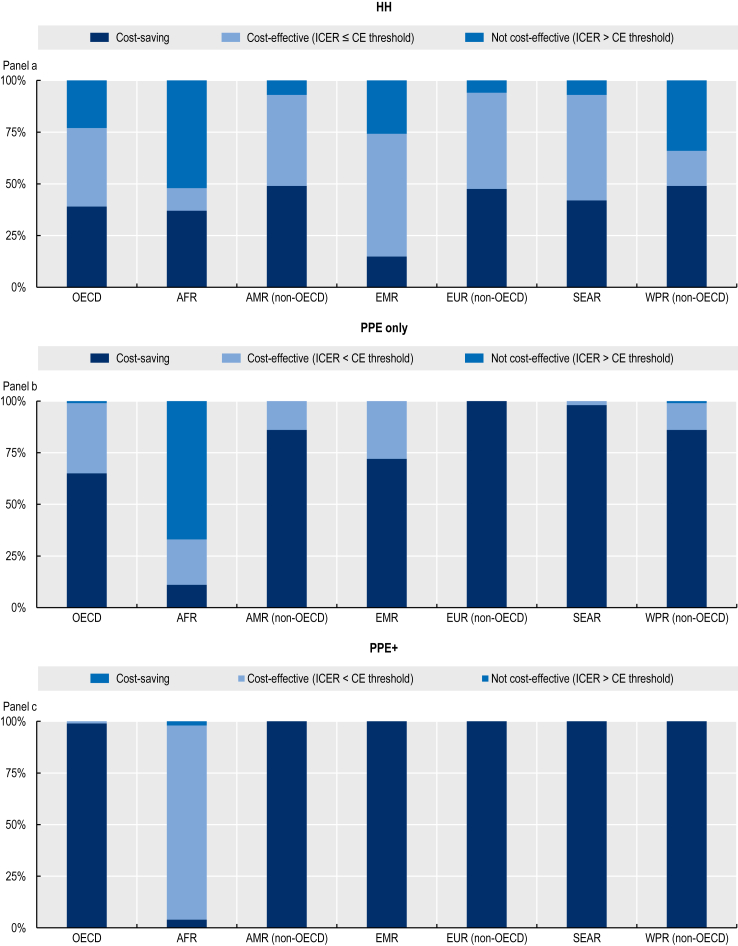
**Probability of cost-effectiveness of three IPC interventions modelled in the study vs. *business-as-usual* scenario**. Modelled IPC interventions are enhancing hand hygiene (HH), increasing access to PPE (PPE only) and increasing access to PPE in combination with IPC training and education (PPE+). In each region, dark blue represents cost-saving interventions, light blue represents cost-effective interventions and medium blue represents not cost-effective interventions. For OECD, the cost-effectiveness ratio was set as 50,000 US$ PPP/DALYs. For other regions, three times that of the gross domestic product per capita was used as the cost-effectiveness threshold as per the WHO-CHOICE guidelines whenever this value was lower than 50,000 US$ PPP/DALYs. When this value exceeded 50,000 US$ PPP/DALYs, 50,000 US$ PPP/DALYs was used as the value of the cost-effectiveness threshold.

The PPE + intervention was estimated to produce the most favourable cost-effectiveness profiles across the three modelled interventions in all regions (Fig. 5). The probability that this intervention would be a cost-saving strategy was nearly 100% in all regions, except in the African region where it was estimated to be 93.5%. Our results suggest that the PPE only intervention might have also offered a cost-saving option. The probability that this intervention would be cost-saving ranged from 64.5 to 99% in all regions, except in the African region. The hand hygiene intervention had the most favourable cost-effectiveness profile in non-OECD countries and the Americas, European and Western Pacific regions where the probability that this was a cost-saving option was estimated to range between 47.8% and 49.1%. Additionally, the probability that hand hygiene was a cost-effective intervention was estimated to be around 60% for non-OECD countries in Europe (see the Appendix for the estimated probabilities).

## Discussion

Our analysis highlights the large health and economic gains that would have been achieved globally by implementing three IPC interventions prior to the start of the COVID-19 pandemic. Our results suggest that estimated gains could have been highest when access to PPE was combined with increased access to IPC education and training. The relatively modest health gains attributable to the hand hygiene intervention are not surprising when considering that SARS-CoV-2 infection is transmitted through multiple routes, but mainly airborne via respiratory droplets and aerosol particles.^26^ Taken together, our results suggest that investments aiming to strengthen IPC capacity, consistent with the WHO IPC core components, should be prioritised to bolster the pandemic response and better prepare for future outbreaks.^8^

Our finding that the PPE only and PPE + interventions could have yielded substantial health and economic gains underscores the importance of securing global supply chains. In the early phases of the outbreak, health systems grappled with significant shortages in PPE, driven largely by an unprecedented surge in demand, coupled with disruptions in global supply chains, a lack of surge plans, and export prohibitions and restrictions. In response, the WHO COVID-19 Supply Chain System was launched in April 2020 and was able to respond to an estimated 45% of PPE needs across 169 countries in the same year.^27^

Our results showing that IPC training and education accentuated the protective effects of PPE underscore the importance of implementing multimodal strategies to strengthen IPC capacity. IPC training and education is an essential element of multimodal strategies and a core component of IPC. However, a 2019 WHO global survey on IPC capacity at health facility level showed that IPC training and education was the core component with the lowest score,^7^ suggesting that many health workers may have been underprepared to appropriately apply IPC measures and use PPE at the beginning of the pandemic. A more recent global WHO survey indicated that IPC training and education remains a major gap in national IPC programmes.^10^

Our data suggest that countries in the African, South-East Asia and Eastern Mediterranean regions are poised to make the greatest health and economics gains by investing in increasing access to PPE and IPC education and training. Cross-regional variations in the potential health and economic gains can be partly explained by differences in the demographic profile of health workers. For example, in the African region, where the health workforce is relatively young,^23^ the PPE + intervention could have averted new infections and hospitalisations, whereas this intervention was not associated with observable changes in the number of ICU admissions and deaths. In OECD countries where the health workforce is older,^23^ the same intervention could have resulted in improvements in all health outcomes studied.

Intervention costs calculated for this analysis were broadly comparable to costs calculated in a previous WHO work on resource needs for an effective response to COVID-19 in 73 LMICs.^4^ The WHO study suggested that US$ 25.23 (between US$ 6.20 and US$ 26.75) per capita would be needed to ensure an effective response to the pandemic across the countries included over a period of 12 weeks, depending on the country income level. It also suggested that 7.3% of costs would be attributable to expenses for upscaling IPC policies, including enhancing hand hygiene and scaling-up access to PPE. Thus, the estimated investment to upscale IPC capacity would be approximately US$ 0.66 to US$ 2.02 per capita. In our study, scaling-up of the three IPC interventions was estimated to have a relatively modest economic cost, on average, US$0.27 to US$1.15 per capita across the seven regions. The small differences between the two analyses can be largely explained by an incomplete match in terms of the scope and focus of the studies, a difference in the length of the study period, and differences in the accounting of certain budget lines. Results on the return on investment and cost-effectiveness of the modelled IPC interventions offered conservative estimates as most costs associated with the implementation of these interventions were budgeted at the beginning of each year, whereas our study only covered the first six months of the pandemic.

Our study differs from earlier studies in important ways. Our analysis covers seven geographic regions, whereas earlier multi-country studies assessing the effectiveness and cost-effectiveness of IPC interventions during COVID-19 focused only on LMICs.^12^ Previous studies also varied in terms of the outcomes measured, reflecting the differences in their objectives. Risko and colleagues focused on premature mortality as a measure of productivity loss, whereas our study extended this analysis by including the impact of absenteeism.^14^ Our analysis also differs in terms of methods and assumptions used. While considering the pandemic, previous studies assumed that the risk of SARS-CoV-2 infection was similar between health workers and the general community.^1^^,^^12^ However, emerging evidence suggests that health workers faced a higher risk of acquiring COVID-19.^2^

Our results should be interpreted with caution. Our estimates of the effectiveness of IPC interventions should not be interpreted as the impact of the IPC interventions actually put in place by countries over the course of the COVID-19 pandemic. Instead, our estimates are intended to allude to what would have happened if the IPC interventions anchored in the WHO IPC core components were already at desirable levels at the outset of the outbreak. They consider demographic characteristics, health service provision and IPC capacity, as well as physical distancing measures in the community in each setting. Our substantive results are robust to several sensitivity checks.

Our study has some limitations. First, the estimates of the effectiveness of the three IPC interventions are surrounded by wide confidence intervals and reflect the current state of evidence in the literature, thus emphasizing the need to generate rigorous scientific. Indeed, we relied on previous systematic reviews and meta-analyses to derive these estimates.^14^^,^^24^ The vast majority of evidence reported in these reviews and meta-analyses were extracted from small-scale and observational studies conducted in the context of SARS-CoV-1, MERS-CoV and at the outset of SARS-CoV-2. These studies consistently pointed to the pressing need to address the existing gaps in data availability and deficiencies in the design of trials measuring the effectiveness of IPC interventions. In our study, we sought to make transparent the methods and assumptions used to derive and present uncertainty in our estimates. Second, our estimates of the effectiveness of IPC interventions do not reflect the broader impact of investing in IPC capacity. For example, they do not consider the protective effects of these interventions on other infectious diseases, including HAIs,^24^ or reducing the transmission of antimicrobial resistance.^28^ Third, our results do not consider the protective effects of the modelled IPC interventions for individuals other than health workers who might be exposed to SARS-CoV-2 in healthcare settings. Fourth, our modelling framework relies on a basic reproduction number to model the spread of the pandemic and does not explicitly model all of the potential transmission patterns of SARS-CoV-2. For example, we did not explicitly model the ways in which health workers may be exposed to SARS-CoV-2 inside or outside of healthcare settings (e.g., exposure to sick family members). We also did not explicitly model the early screening, detection and surveillance capacity for COVID-19 in healthcare settings and in the community. Yet, findings from previous studies suggest that improving early screening, detection and surveillance capacity could help reduce the spread of SARS-CoV-2 infections and possibly influence the effectiveness of the modelled interventions.^16^ Considering this, we investigated the sensitivity of our findings under different pandemic scenarios using various basic reproduction numbers ranging from 1.5 to 5 (Appendix pp. 45–47). Combined, findings from these analyses suggested that there is an inverse relationship between the effectiveness of the three modelled interventions and the basic reproductive number, implying that a higher reproduction number is likely to hinder the protective effects of these interventions at the population level. Conversely, a lower reproduction number can help accentuate the protective effects of the modelled IPC interventions, resulting in lower number of infections averted among health workers. Fifth, we relied on the evidence available in the literature to select the key model parameters and assumptions. For example, we assumed that recovered individuals acquired immunity and could not be re-infected in the remainder of the simulation. The evidence on immunity acquired after recovery from SARS-CoV-2 infection remains unclear, although a growing number of studies point out that immunity may wane over time, with estimates ranging from 3 to 6 months after the initial infection.^29^ These estimates fall within the timeframe of our analysis. We used data from the 2019 WHO IPC Self-Assessment Framework to estimate the baseline level of coverage for each intervention. While this global survey uses a standardised validated tool to assess IPC capacity at the facility level, the precision of our estimates of the baseline coverage of each modelled intervention may be prone to self-report bias. Finally, we did not attempt to quantify the potential impact of lower health worker productivity on population health outcomes. However, emerging studies demonstrate that many health workers report longer work hours, experience adverse mental health effects,^2^ and risk burnout in order to keep up with the unprecedented rise in the demand for care.^30^

In conclusion, enhancing IPC capacity in healthcare settings has a great potential for averting adverse health outcomes among health workers, while yielding substantial savings. Building robust IPC capacity and programmes will require deliberate investments and should be prioritised as a critical strategy to reduce the adverse health and economic effects of the COVID-19 pandemic and to ensure that health systems across the globe are adequately prepared for future outbreaks.

## Contributors

BA, AC, MC, and EO led the study design; BA, AC, MC, and OE provided data sources to be included in the model. AC, AL and EO directly accessed and verified the underlying data. AL, EO and MC developed the statistical analysis plan. AL and EO performed data analysis and generated tables and figures. EO and MC led the manuscript writing. BA and AC substantially contributed to the manuscript writing. All authors were involved in data interpretation, reviewed drafts of the manuscript, and provided critical input. All authors approved the final version of the manuscript.

## Data sharing statement

All data used in this study are publicly available except those extracted from the 2019 WHO IPC Self-Assessment Framework. Data from the 2019 WHO IPC Self-Assessment Framework can be requested from Dr Sara Tomczyk (tomczyks@rki.de), who is the corresponding author of the original research article published in Lancet Infectious Diseases.

## Declaration of interests

MC reports that the OECD programme of work on public health is supported by statutory and voluntary contributions provided by OECD member countries’ governmental institutions, as well as other international organisations.
