# Supplementary material for: Estimating the return on investment of selected infection prevention and control interventions in healthcare settings for preparing against novel respiratory viruses: modelling the experience from SARS-CoV-2 among health workers
Source: eClinicalMedicine. 2024 Jan 5;68:102388. doi: 10.1016/j.eclinm.2023.102388 (PMC10809104; doi:10.1016/j.eclinm.2023.102388)
Supplement: Appendix [file mmc1.pdf]

Estimating the return on investment of selected infection prevention and control interventions in healthcare settings for preparing against novel respiratory viruses: modelling the experience from SARS-CoV-2 among health workers

Appendix

Table A.1. shows the list of countries included in the analysis by WHO-OECD regions.

Table A.1. World Health Organisation (WHO)-Organization for Economic Co-operation and Development (OECD) regional designations*

Country	ISO3	WHO-OECD region	Country	ISO3	WHO-OECD region
Algeria	DZA	AFR	Afghanistan	AFG	EMR
Angola	AGO	AFR	Bahrain	BHR	EMR
Benin	BEN	AFR	Djibouti	DJI	EMR
Botswana	BWA	AFR	Egypt, Arab Rep.	EGY	EMR
Burkina Faso	BFA	AFR	Iran, Islamic Rep.	IRN	EMR
Burundi	BDI	AFR	Iraq	IRQ	EMR
Cabo Verde	CPV	AFR	Jordan	JOR	EMR
Cameroon	CMR	AFR	Kuwait	KWT	EMR
Central African Republic	CAF	AFR	Lebanon	LBN	EMR
Chad	TCD	AFR	Libya	LBY	EMR
Comoros	COM	AFR	Morocco	MAR	EMR
Congo, Dem. Rep.	COD	AFR	Oman	OMN	EMR
Congo, Rep.	COG	AFR	Pakistan	PAK	EMR
Cote d'Ivoire	CIV	AFR	Qatar	QAT	EMR
Equatorial Guinea	GNQ	AFR	Saudi Arabia	SAU	EMR
Eritrea	ERI	AFR	Somalia	SOM	EMR
Eswatini	SWZ	AFR	Sudan	SDN	EMR
Ethiopia	ETH	AFR	Syrian Arab Republic	SYR	EMR
Gabon	GAB	AFR	Tunisia	TUN	EMR
Gambia, The	GMB	AFR	United Arab Emirates	ARE	EMR
Ghana	GHA	AFR	Yemen, Rep.	YEM	EMR
Guinea	GIN	AFR	Antigua and Barbuda	ATG	Non-OECD AMR
Guinea-Bissau	GNB	AFR	Argentina	ARG	Non-OECD AMR
Kenya	KEN	AFR	Bahamas, The	BHS	Non-OECD AMR
Lesotho	LSO	AFR	Barbados	BRB	Non-OECD AMR
Liberia	LBR	AFR	Belize	BLZ	Non-OECD AMR

Madagascar	MDG	AFR	Bolivia	BOL	Non-OECD AMR
Malawi	MWI	AFR	Brazil	BRA	Non-OECD AMR
Mali	MLI	AFR	Cuba	CUB	Non-OECD AMR
Mauritania	MRT	AFR	Dominica	DMA	Non-OECD AMR
Mauritius	MUS	AFR	Dominican Republic	DOM	Non-OECD AMR
Mozambique	MOZ	AFR	Ecuador	ECU	Non-OECD AMR
Namibia	NAM	AFR	El Salvador	SLV	Non-OECD AMR
Niger	NER	AFR	Grenada	GRD	Non-OECD AMR
Nigeria	NGA	AFR	Guatemala	GTM	Non-OECD AMR
Rwanda	RWA	AFR	Guyana	GUY	Non-OECD AMR
Sao Tome and Principe	STP	AFR	Haiti	HTI	Non-OECD AMR
Senegal	SEN	AFR	Honduras	HND	Non-OECD AMR
Seychelles	SYC	AFR	Jamaica	JAM	Non-OECD AMR
Sierra Leone	SLE	AFR	Nicaragua	NIC	Non-OECD AMR
South Africa	ZAF	AFR	Panama	PAN	Non-OECD AMR
South Sudan	SSD	AFR	Paraguay	PRY	Non-OECD AMR
Tanzania	TZA	AFR	Peru	PER	Non-OECD AMR
Togo	TGO	AFR	St. Kitts and Nevis	KNA	Non-OECD AMR
Uganda	UGA	AFR	St. Lucia	LCA	Non-OECD AMR
Zambia	ZMB	AFR	St. Vincent and the Grenadines	VCT	Non-OECD AMR
Zimbabwe	ZWE	AFR	Suriname	SUR	Non-OECD AMR
Trinidad and Tobago	TTO	Non-OECD AMR	Vietnam	VNM	Non-OECD WPR
Uruguay	URY	Non-OECD AMR	Australia	AUS	OECD
Venezuela, RB	VEN	Non-OECD AMR	Austria	AUT	OECD
Albania	ALB	Non-OECD EUR	Belgium	BEL	OECD
Andorra	AND	Non-OECD EUR	Canada	CAN	OECD
Armenia	ARM	Non-OECD EUR	Chile	CHL	OECD
Azerbaijan	AZE	Non-OECD EUR	Colombia	COL	OECD

Belarus	BLR	Non-OECD EUR	Costa Rica	CRI	OECD
Bosnia and Herzegovina	BIH	Non-OECD EUR	Czech Republic	CZE	OECD
Bulgaria	BGR	Non-OECD EUR	Denmark	DNK	OECD
Croatia	HRV	Non-OECD EUR	Estonia	EST	OECD
Cyprus	CYP	Non-OECD EUR	Finland	FIN	OECD
Georgia	GEO	Non-OECD EUR	France	FRA	OECD
Kazakhstan	KAZ	Non-OECD EUR	Germany	DEU	OECD
Kyrgyz Republic	KGZ	Non-OECD EUR	Greece	GRC	OECD
Malta	MLT	Non-OECD EUR	Hungary	HUN	OECD
Moldova	MDA	Non-OECD EUR	Iceland	ISL	OECD
Monaco	MCO	Non-OECD EUR	Ireland	IRL	OECD
Montenegro	MNE	Non-OECD EUR	Israel	ISR	OECD
North Macedonia	MKD	Non-OECD EUR	Italy	ITA	OECD
Romania	ROU	Non-OECD EUR	Japan	JPN	OECD
Russian Federation	RUS	Non-OECD EUR	Korea, Rep.	KOR	OECD
San Marino	SMR	Non-OECD EUR	Latvia	LVA	OECD
Serbia	SRB	Non-OECD EUR	Lithuania	LTU	OECD
Tajikistan	TJK	Non-OECD EUR	Luxembourg	LUX	OECD
Turkmenistan	TKM	Non-OECD EUR	Mexico	MEX	OECD
Ukraine	UKR	Non-OECD EUR	Netherlands	NLD	OECD
Uzbekistan	UZB	Non-OECD EUR	New Zealand	NZL	OECD
Brunei Darussalam	BRN	Non-OECD WPR	Norway	NOR	OECD
Cambodia	KHM	Non-OECD WPR	Poland	POL	OECD
China	CHN	Non-OECD WPR	Portugal	PRT	OECD
Cook Islands	COK	Non-OECD WPR	Slovak Republic	SVK	OECD
Fiji	FJI	Non-OECD WPR	Slovenia	SVN	OECD
Kiribati	KIR	Non-OECD WPR	Spain	ESP	OECD
Lao PDR	LAO	Non-OECD WPR	Sweden	SWE	OECD

Malaysia	MYS	Non-OECD WPR	Switzerland	CHE	OECD
Marshall Islands	MHL	Non-OECD WPR	Turkey	TUR	OECD
Micronesia, Fed. Sts.	FSM	Non-OECD WPR	United Kingdom	GBR	OECD
Mongolia	MNG	Non-OECD WPR	United States	USA	OECD
Nauru	NRU	Non-OECD WPR	Bangladesh	BGD	SEAR
Niue	NIU	Non-OECD WPR	Bhutan	BTN	SEAR
Palau	PLW	Non-OECD WPR	India	IND	SEAR
Papua New Guinea	PNG	Non-OECD WPR	Indonesia	IDN	SEAR
Philippines	PHL	Non-OECD WPR	Maldives	MDV	SEAR
Samoa	WSM	Non-OECD WPR	Myanmar	MMR	SEAR
Singapore	SGP	Non-OECD WPR	Nepal	NPL	SEAR
Solomon Islands	SLB	Non-OECD WPR	Sri Lanka	LKA	SEAR
Tonga	TON	Non-OECD WPR	Thailand	THA	SEAR
Tuvalu	TUV	Non-OECD WPR	Timor-Leste	TLS	SEAR
Vanuatu	VUT	Non-OECD WPR			

* List of countries and associated WHO-OECD regional designations included in the analysis. ISO3 codes were obtained from the World Bank.

Modelling the severity of the progression of SARS-CoV-2

Similar to earlier studies,[3] the SPHeP COVID-19 model uses the infection severity table published by Salje and colleagues as baseline data (2020).[4] This study provides three different estimates of the likelihood of the progression of SARS-CoV-2 by gender and age group: likelihood of hospitalisation (P_H); likelihood of admission to the ICU if hospitalised (P_{ICU}); and likelihood of death if hospitalised (P_D). These data are then transformed using the methodology described below to provide age- and gender-specific estimates for the four following parameters:

- P_H: likelihood of hospitalisation
- P_{ICU}: likelihood of admission to the ICU (among hospitalised patients)
- $P_{D|\overline{ICU}}$: likelihood of death among hospitalised non-ICU patients
- $P_{D|ICU}$: likelihood of death among ICU patients

The original estimates are first interpolated by age, assuming a flat rate after 90 years of age. Similar to Walker and colleagues,[3] the likelihood of death in the ICU is then computed by aggregating the likelihood of admission to the ICU if hospitalised and the likelihood of death if hospitalised, assuming that 80% of deaths occur in the ICU. Nevertheless, this assumption is not plausible (and denied by data) at older ages as access to the ICU decreases. To adjust for this, we made an additional assumption that mortality in the ICU cannot exceed 60%. Our approach yields the following likelihood of death among ICU patients and the likelihood of deaths among hospitalised, non-ICU patients:

$$P_{D|ICU} = \min\left(\frac{80\% \cdot P_D}{P_{ICU}}, 60\%\right)$$

$$P_{D|\overline{ICU}} = \frac{P_D - P_{D|ICU} \cdot P_{ICU}}{1 - P_{ICU}}$$

Fig. A.1. summarises the likelihood of hospitalisation, likelihood of ICU admission among hospitalised patients, likelihood of death among hospitalised, non-ICU patients, and the likelihood of death among ICU patients by age categories for females and males.

Fig A.1. Severity of disease progression of SARS-CoV-2.

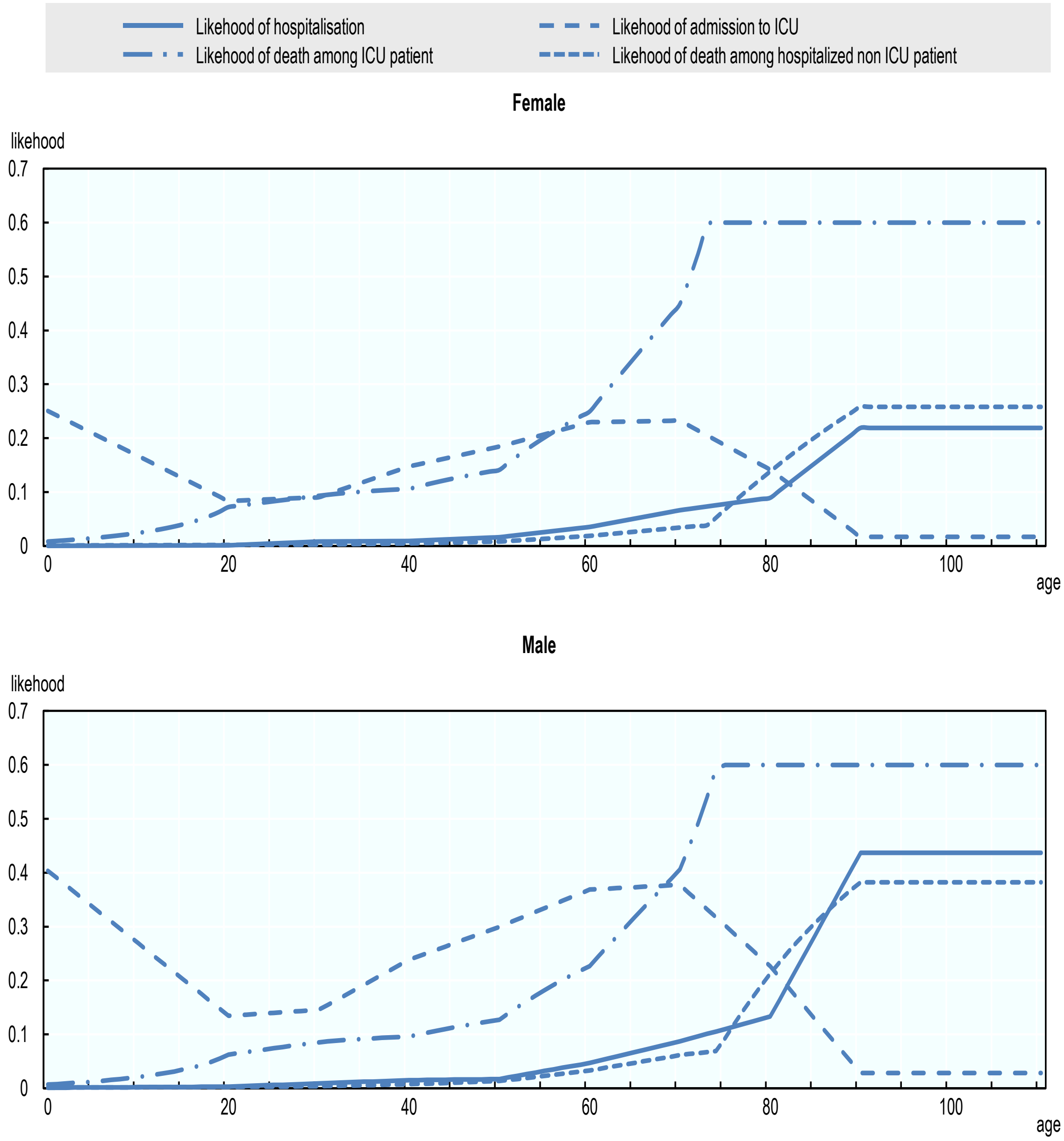

Estimating the risk of SARS-CoV-2 infections among health workers compared to the general community

A growing body of evidence suggests that health workers face a greater risk of SARS-CoV-2 infections compared to the general community.[18,19] Recognizing the emerging evidence, the OECD SPHeP COVID-19 model incorporates estimates on the relative risk of infections among health workers in each WHO-OECD compared to the general community in that region.

Analysis sample and data

We extracted country-aggregated data on the number of SARS-CoV-2 infections from the WHO COVID-19 Surveillance Database covering the period from January 1st to July 30th, 2020. This database was developed as part of the emergency response to strengthen public health surveillance over the course of the pandemic. The number of new SARS-CoV-2 infections among health workers and in the general community are reported to the database by country officials on a daily basis. People who test positive for SARS-CoV-2 are considered as infected, regardless of whether they experience any symptoms. The database makes use of a comprehensive definition of health workers that encompass not only those directly involved in caring for COVID-19 patients, but also those who could have come into contact with a patient's biological fluid/respiratory secretions, potentially contaminated objects, and environmental surfaces. Health worker categories include allied and auxiliary health workers, such as cleaning and laundry personnel, radiology physicians and technicians, administrative staff, phlebotomists, respiratory therapists, nutritionists, social workers, physical therapists, laboratory personnel, admission/reception clerks, patient transporters and

catering staff, as well as other personnel categories as defined by countries in order to adapt to the local context.

As shown in Table A.2., our analysis sample comprised 53 countries that reported data in 2020 between January 1st to July 30th on the number of SARS-CoV-2 infections among health workers and the general community to gauge the level of preparedness in terms of IPC capacity at the outset of the pandemic. We excluded countries that did not report SARS-CoV-2 infections among health workers, as well as countries that reported data for less than 20% of the analysis period in order to ensure that our study sample was drawn from countries that routinely provided information to the WHO COVID-19 Detailed Surveillance Database.

Table A.2. List of 53 countries included for calculation of the relative risk of SARS-CoV-2 infection in the general public versus health workers.

Country code	Country	Region
DZA	Algeria	AFR
AGO	Angola	AFR
COD	Democratic Republic of the Congo	AFR
SWZ	Eswatini	AFR
ETH	Ethiopia	AFR
KEN	Kenya	AFR
MWI	Malawi	AFR
MOZ	Mozambique	AFR
NAM	Namibia	AFR
RWA	Rwanda	AFR
SLE	Sierra Leone	AFR
ZAF	South Africa	AFR
UGA	Uganda	AFR
ZMB	Zambia	AFR
ZWE	Zimbabwe	AFR
IRQ	Iraq	EMR
JOR	Jordan	EMR
PAK	Pakistan	EMR
QAT	Qatar	EMR
SYR	Syrian Arab Republic	EMR
TUN	Tunisia	EMR
ARG	Argentina	Non-OECD AMR
BHS	Bahamas	Non-OECD AMR
BRA	Brazil	Non-OECD AMR

DOM	Dominican Republic	Non-OECD AMR
ECU	Ecuador	Non-OECD AMR
GTM	Guatemala	Non-OECD AMR
JAM	Jamaica	Non-OECD AMR
VEN	Venezuela (Bolivarian Republic of)	Non-OECD AMR
CYP	Cyprus	Non-OECD EUR
MLT	Malta	Non-OECD EUR
ROU	Romania	Non-OECD EUR
AUT	Austria	OECD
CAN	Canada	OECD
CHL	Chile	OECD
CRI	Costa Rica	OECD
CZE	Czechia	OECD
EST	Estonia	OECD
FIN	Finland	OECD
HUN	Hungary	OECD
IRL	Ireland	OECD
ITA	Italy	OECD
LVA	Latvia	OECD
LTU	Lithuania	OECD
LUX	Luxembourg	OECD
MEX	Mexico	OECD
NLD	Netherlands	OECD
NOR	Norway	OECD
POL	Poland	OECD
PRT	Portugal	OECD
SVK	Slovakia	OECD
SWE	Sweden	OECD
USA	United States of America	OECD

We obtained data on the total number of health workers in each country from the WHO National Health Workforce Accounts Data Portal.[20] This dataset collates annualised information on the number of health workers by 40 different categories from national reports, censuses, labour force statistics and other national and regional administrative resources covering the period from 2014 to 2019. We calculated the total number of health workers in each country as the sum of all different categories of health workers for the most recent year for which data were available.

Outcome and control variables

We calculated the risk of SARS-CoV-2 infections among health workers relative to the general community in several steps. First, we separately calculated the total number of

people with SARS-CoV-2 infections among health workers and in the general community. The general community refers to the population of each country except for health workers. Data on the population size of each country were extracted from the United Nations Population Division estimates for the year 2020.[21] Next, we derived the proportion of health workers and general community who were reported having a SARS-CoV-2 infection. Finally, we calculated the risk of SARS-CoV-2 infections in health workers compared to the general community by dividing the proportion of health workers with SARS-CoV-2 infections by the proportion of infected people in the general community.

We adjusted the risk of SARS-CoV-2 infections among health workers compared to the general community as a function of country-aggregated covariates to capture demographic and socioeconomic characteristics according to the proportion of population living in rural areas, the proportion of population aged 65 years and older, the share of gross domestic product (GDP) spent on health, and per capita GDP, as done by Walker and colleagues,[3] while deriving estimates to feed into the COVID-19 model developed by Imperial College London (UK). We extracted these data for the year 2020 from the World Development Indicators Database.[22]

Statistical analysis

In our country-level dataset, data on the proportion of population living in rural areas and the proportion of population aged 65 years and older were complete, whereas 8% of countries lacked data on GDP per capita. Data on the share of GDP spent on health were not available for any countries for the year 2020. For this variable, we used the

2018 data for the remainder of the analysis, the most recent year for which data were available. Only 2% of countries were missing data on this covariate.

To fill in the missing values, we used multiple imputation based on the Amelia II algorithm.[23] This method assumes that the complete data are jointly distributed in accordance with a multivariate normal distribution and that the data are missing at random, which allowed us to impute the missing observations based on the correlations observed across all covariates. The method imputes *m* values defined by the researchers for each missing data point in the dataset, based on the data observed within the same dataset, creating *m* completed datasets. Across the completed datasets, the observed values of each covariate remain the same while the missing values are replaced with the imputed values. In our analysis, we imputed all country-aggregated data 150 times and a 1% ridge prior was used to improve stability. Before applying the algorithm, we log transformed our outcome variable and GDP per capita due to the skewed distribution of these variables. We ran over-imputation diagnostics to ascertain the fit of the imputation model as recommended by the Amelia documentation.[23]

After confirming the goodness of fit of the imputed variables, we carried out the remainder of the analyses using the 150 completed datasets. We estimated a linear model to predict the risk of SARS-CoV-2 infections among health workers compared to the public as shown in the equation (1) below:

$$\log(Y_c) = \gamma X_c + \varphi_r + \varepsilon_c \quad (1)$$

Equation (1) yields the average risk of SARS-CoV-2 infections among health workers compared to the general community in each country, adjusted for demographic and socioeconomic characteristics: $\log(Y_c)$ denotes the natural logarithm of the risk of SARS-CoV-2 infections among health workers compared to the general community in the country; *c*. $\mathrm{X_c}$ is the vector of country-level controls; φ_r represents the WHO-OECD region fixed effects to take into account time-invariant differences across regions that may correlate with the outcome variable; ε_c denotes the residual. To derive the WHO-OECD regional estimates, we aggregated our country-level results, while adjusting for the population size of each country. For countries that reported no data to the WHO Surveillance Database, we used estimates generated and based on the World Bank income strata.[22]

Results

As shown in Table A.3., the African region had the highest risk of COVID-19 infections among health workers compared to the general community, whereas the OECD region had the lowest risk (health workers to community infection ratio, 21.68 and 4.86, respectively). Health workers in other regions also faced a greater risk of SARS-CoV-2 infections. In the Eastern Mediterranean and South-East Asia regions, health workers faced a 16.37- and 15.66-fold increase, respectively, in the risk of SARS-CoV-2 infection compared to the general community. In non-OECD countries within the European, Western Pacific and the Americas regions, health workers faced an increased risk of SARS-CoV-2 infections compared to the general community of 10.29, 8.63 and 8.45, respectively.

Table A.3. Risk of SARS-CoV-2 infections and health workers compared to the general community.

WHO-OECD regions	Estimate
OECD	4.86
non-OECD countries in the Americas region	8.45
non-OECD European region countries	10.29
non-OECD Western Pacific region countries	8.63
Eastern Mediterranean region countries	16.37
African region countries	21.68
South-East Asia region countries	15.66

Limitations

In our analysis, we considered this difference in the risk of SARS-CoV-2 infections by using data from the WHO COVID-19 Detailed Surveillance Database. This database was previously used by the WHO to estimate mortality among health workers from January 1, 2020, to May 31, 2021.[24] Unlike the WHO analysis, we used this database only to estimate the differences in the risk of SARS-CoV-2 infections among health workers compared to the general community and we estimated the impact of the pandemic on health outcomes using parameter values extracted from the available literature to the best extent possible.

One concern with using data from the WHO COVID-19 Detailed Surveillance Database relates to a potential underreporting of new SARS-CoV-2 infections. Several factors may drive underreporting. If health workers are more likely to get tested compared to the general public, estimates generated using this dataset may overstate the risk of infection among health workers. Alternatively, new SARS-CoV-2 infections among health workers may be underreported if administrative data does not always record that the infected individual is a health worker, resulting in an understatement of the risk of infection among this population. Taken together, the challenges around using these

administrative data may have rendered our estimates imprecise, but we are unable to disentangle the direction and the magnitude of the bias. In our study, we used data from countries with the most complete information and compared our estimates against results generated by single-country studies. With these caveats in mind, we argue that the WHO COVID-19 Surveillance Database offers the most comprehensive source of data to assess differences in the risk of SARS-CoV-2 infections among health workers compared to the general population across different geographic regions.

Model outputs

Health impacts

The number of disability-adjusted life years (DALYs) attributable to COVID-19 were quantified as a composite of years of life lost (YLLs) due to premature mortality and years of healthy life lost due to disability (YLDs). YLLs were quantified by multiplying the number of attributable deaths by the difference in age at attributable death and age-specific life expectancy in each country. We extracted standard life expectancy tables from the United Nations World Population Project and Human Mortality database.

YLDs were calculated by multiplying the length of time lived with a health state by the corresponding disability weights for that health state, as shown in Table A.4. Since no country had a prior history of SARS-CoV-2 infection, there are currently no global or regional estimates for disability weights associated with COVID-19. To date, studies that aimed at calculating the magnitude of health loss attributable to COVID-19 have relied on country-specific disability weights for other health conditions[5] or used disability weights generated by the Global Burden of Disease (GBD) study for lower respiratory

infections.[6,7] The European Burden of Disease Network also suggested to use disability weights from the GBD study in a recently published protocol for country studies.[8]

In our study, we used disability weights generated by the GBD study for lower respiratory infection whenever possible, as shown in Table A.4. and Fig. A.2. Disability weights generated by Haagsma and colleagues (2015) for lower respiratory infection were applied to critical COVID-19 cases, which included hospitalised ICU patients, post-ICU long-term sequelae for critical COVID-19 cases, post-acute phase (i.e., fatigue, emotional lability and insomnia) for severe and critical cases, and 'long' COVID as the GBD study does not provide estimates for these health states.[9] Table A.5. shows the share of symptomatic patients experiencing long-term consequences by age categories, though it should be noted that the existing evidence on the estimated prevalence of patients experiencing long-term consequences of COVID-19 point to a huge variation reflecting the differences in study settings, case definitions and methods of analyses.

Table A.4. Health states, disability weights, and duration of health state for COVID-19 estimates.

Health state	Disability weight (95% uncertainty interval)
Asymptomatic	0
Mild/moderate	0.051 (0.032-0.074)[10]
Hospitalised, non-ICU patient	0.125 (0.104-0.152)[10]
Hospitalised, ICU patient	0.655 (0.579-727)[9]
Post-acute consequences (fatigue, emotional lability, insomnia)	0.217 (0.179-0.251)[9]
Post-ICU long-term sequelae	0.217 (0.179-0.251)[9]
Long-term consequences (long-COVID)	0.185 (0.154-0.223)[9]

Table A.5. Percentage of symptomatic patients experiencing long-term consequences by age categories. Office for National Statistics, 2021.[11]

Age categories (years)	% of people experiencing long-term consequences
2-11	0.11
12-16	0.40
17-24	0.91
25-34	1.13
35-49	1.58
50-69	1.58
≥70	0.67

Fig. A.2. Health states associated with COVID-19. D, duration; d, day, DW, disability weight; EL, emotional lability; F, fatigue; I, insomnia; RLE, remaining life expectancy; y, year

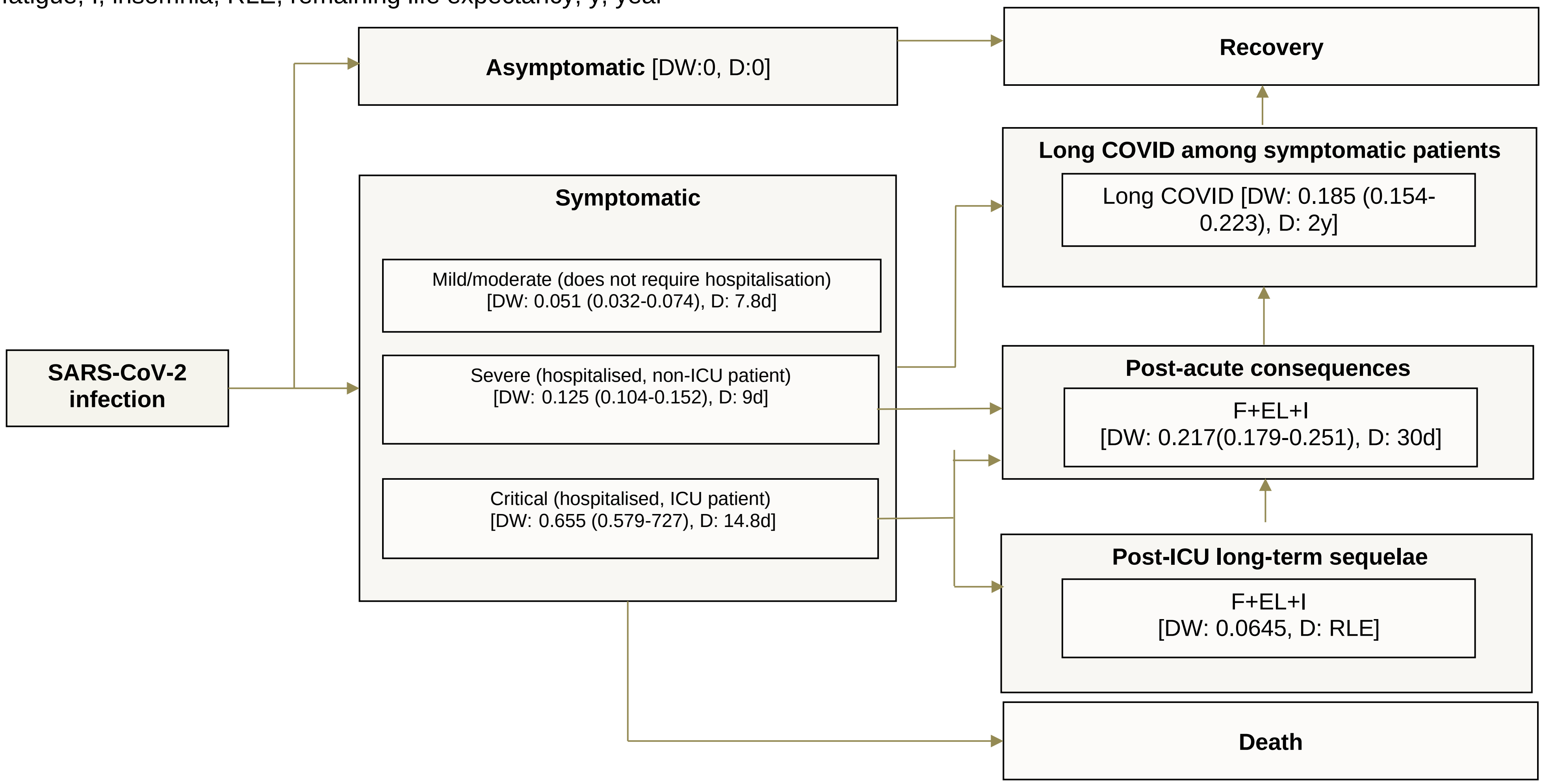

Economic impact

The OECD SPHeP COVID-19 model quantifies the economic impact of healthcare-associated SARS-CoV-2 infection across two dimensions: 1) cost of medical treatment and 2) productivity loss due to absence from work. Cost of medical care refers to expenses incurred due to treating infected health workers. Medical treatment covers both inpatient care and ICU care. The cost of medical treatment was derived by multiplying the number of attributable hospitalisations by country-specific cost estimates of medical treatment. For input costs, country-specific estimates for the cost of inpatient care per person per day was used and adjusted for the year 2020. Throughout the analysis, all costs are expressed in US$ for the year 2020 and adjusted for purchasing power parity (PPP) to account for country-level differences in prices.

Productivity loss refers to reduced labour output among health workers due to absence from work associated with ill health and is calculated as a function of salary loss.[12,13] Productivity losses are measured across three dimensions: 1) quarantine policies; 2) attributable hospitalisation and recovery after discharge from hospital; and 3) premature mortality. Of note, a growing body of evidence demonstrates that some patients continue to present symptoms after recovering from an initial SARS-CoV-2 infection, a phenomenon referred to as ‘long’ COVID.[14] It is plausible that the productivity of health workers suffering from long COVID may be reduced, compared to their colleagues who do not experience any symptoms. While it is desirable to quantify potential loss of productivity due to long COVID, the dearth of empirical evidence in the existing literature makes it difficult to model the potential impact of attributable presenteeism.

Quarantine policies

In many countries, quarantine policies were put in place to ensure that health workers infected with SARS-CoV-2 remain in isolation, even if they did not require treatment in a health facility. We assumed that health workers diagnosed with COVID-19 cannot be present at their place of work for a period of 10 days in line with WHO quarantine guidelines. We quantified the cost of quarantine by multiplying the number of workdays missed by the average daily wage of health workers in each country. For this analysis, two data sources were used. First, we applied the current WHO guidelines on safe return to work for health workers dated 16 October 2020 (see Table A.6. for further details). This approach was taken as it was not possible to extract information on the return-to-work criteria for health workers used in each country included in the analysis sample. To validate our approach, we also conducted a desk review of the return-to-work guidelines for health workers produced by other international bodies and countries covering the study period. As shown in Table A.6., this analysis revealed no major differences with the existing WHO guidelines.

Table A.6. Desk review of sample guidelines for return to work for health workers.

Agency	Date issued	Guidelines
World Health Organization	October 30, 2020	Symptomatic patients can be released from isolation:<br>• 10 days after symptom onset, plus at least 3 additional days without (including without fever and without respiratory symptoms).<br>Asymptomatic patients can be released from isolation:<br>• 10 days after they first tested positive
United States Centres for Disease Control and Prevention (US-CDC)	February 16, 2021	Health workers with mild-to-moderate illness who are not severely immunocompromised:<br>• at least 10 days have passed since symptoms first appeared and<br>• at least 24 hours have passed since last fever without the use of fever-reducing medications and<br>• symptoms (e.g., cough, shortness of breath) have improved.<br>Health workers who were asymptomatic throughout their infection and are not severely immunocompromised:<br>• at least 10 days have passed since the date of their first positive viral diagnostic test.<br>Health workers with severe-to-critical illness or who are severely immunocompromised:<br>• at least 10 days and up to 20 days have passed since symptoms first appeared, and<br>• at least 24 hours have passed since the last fever without the use of fever-reducing medications and symptoms (e.g., cough, shortness of breath) have improved following consultation with infection control experts.
Public Health England	January 28, 2021	Symptomatic staff who test positive for SARS-CoV-2 or who have an inconclusive test result, and symptomatic staff who have not had a test can:<br>• return to work no earlier than 10 days from symptom onset, with the condition that a clinical improvement has been observed and the health worker has been afebrile for 48 hours and medically fit to return.<br>Asymptomatic staff:<br>• staff who test positive for SARS-CoV-2 (either by a polymerase chain reaction (PCR) or lateral flow device test) and who were asymptomatic at the time of the test must self-isolate for 10 full days following the date of the test. If they remain well, they can return to work after their isolation period. If, during the 10 days' isolation, they develop symptoms, they must self-isolate for 10 days from the day of symptom onset.
British Columbia Ministry of	January 26, 2021	Health workers with laboratory-confirmed COVID-19 and mild-to-moderate illness that can be managed at home:<br>• self-isolate for 10 days from the onset of symptoms.<br>Health workers with severe illness or severely immunocompromised:

Health (Canada)		• self-isolate for 20 days from the onset of symptoms.
European Centre for Disease Prevention and Control (ECDC)	October 16, 2020	Asymptomatic people who have had a positive SARS-CoV-2 test:<br>• self-isolate for 10 days from the date the sample was taken.<br><br>Mild-to-moderate COVID-19 cases (probable or confirmed):<br>• resolution of fever for at least 3 days and clinical improvement of symptoms other than fever AND<br>• 10 days after the onset of symptoms OR<br>• two consecutive, negative SARS-CoV-2 reverse transcription (RT)-PCR tests in a 24-hour interval from respiratory specimens<br>Severe COVID-19 cases (probable or confirmed):<br>• resolution of fever for at 3 three days and clinical improvement of symptoms other than fever AND<br>• minimum 14 and up to 20 days after the onset of symptoms OR<br>• two consecutive, negative SARS-CoV-2 RT-PCR tests in a 24-hour interval from respiratory specimens.
Korean Centre for Disease Prevention and Control<br><br>(Republic of Korea)	June 25, 2020	Asymptomatic people who have had a positive SARS-CoV-2 test:<br>• discharge if patient does not exhibit any clinical symptoms for 10 days upon confirmation. By the testing criteria, the patient should test negative on PCR tests twice in a row with at least a 24-hour interval after 7 days upon confirmation.<br>Symptomatic people who have had a positive SARS-CoV-2 test:<br>• by clinical criteria, a person should not exhibit fever without taking fever reducers and show improvements in clinical symptoms for at least 72 hours after 10 days upon onset OR<br>• by testing criteria, a person should not exhibit fever without taking fever reducers and show improvements in clinical symptoms after 7 days upon onset. Thereafter, the person should test negative on PCR tests twice in a row with at least a 24-hour interval.

The other data source needed for this analysis was the average daily wage of health workers. We obtained the country-level annual wage estimates of health workers from the WHO for the year 2010 and inflated these estimates for the year 2020. A more detailed description of the WHO methodology to derive these wage estimates are provided elsewhere.[15]

Cost of absence from work due to attributable hospitalisations

This analysis considers both the length of hospital stay (i.e., inpatient care and ICU care) and return to work after critical illness. For hospitalised individuals, we derived this by multiplying the average daily wage by the length of hospital stay and the number of days for return to work after hospital discharge. This approach was preferred as a growing body of evidence highlights that it may take time for patients who suffered from a critical illness to return to work.[16,17] For instance, one recent systematic review and meta-analysis calculated that only about 33% of people who suffered a critical illness returned to employment at 3 months.[16] This study estimated that return to employment increased to about 55% and 56% at 6 and 12 months, respectively. For individuals who may be discharged from a hospital earlier than the quarantine timeframe recommended by WHO, it is assumed that the quarantine period is completed at home.

Premature mortality

Losses in productivity due to quarantine policies and absence from work due to hospitalisation and recovery after discharge from hospital can be considered as temporary losses in productivity due to ill health. The third outcome aims to quantify permanent productivity loss associated with premature mortality among health workers.

We calculated permanent productivity loss by deriving the number of YLLs minus a retirement age of 65 years.

Key design features and model parameters for the IPC interventions modelled in the study

Our study models the effectiveness and cost effectiveness of three IPC interventions that can plausibly reduce the transmission of SARS-CoV-2 infection in healthcare settings. The design features are consistent with current WHO guidelines to the best extent possible, which may not necessarily be reflective of the IPC interventions already in place in each country. The remainder of this section details the methodology by which key model parameters were selected.

Intervention 1: Enhance hand hygiene practices

Overview of the literature

In the context of the ongoing outbreak, relatively little is known about the protective effects of hand hygiene practices in healthcare settings. To date, only a handful of studies have investigated the relationship between hand hygiene and the risk of SARS-CoV-2 infection transmission in health workers. Findings from these studies should be interpreted with caution due to important methodological limitations. One retrospective cohort study from China suggested that suboptimal hand hygiene practices before and after contact with patients was associated with an increased risk of SARS-CoV-2 infection.[27] Similarly, a cross-sectional study from Egypt concluded that adherence to proper hand hygiene guidelines among health professionals in a gastroenterological department was associated with a lower risk of transmission.[28] Conversely, a cross-

sectional study from Bangladesh suggested that compliance with hand hygiene practices during routine patient care moments (eg, during patient care, after body-fluid exposure) had no discernible effect on the risk of SARS-CoV-2 infection.[29]

Several systematic reviews shed light on the role of hand hygiene practices in the context of previous outbreaks. One Cochrane review found that hand hygiene practices were effective in interrupting the spread of SARS-CoV-1 during the 2003 epidemic in healthcare settings (odds ratio [OR] 0.54; 95% CI 0.44-0.67).[30] More recently, a living rapid review conducted by Chou and colleagues[31] (2022) examined evidence from the ongoing outbreak, as well as previous health emergencies including SARS-CoV-1 and Middle East respiratory syndrome coronavirus (MERS-CoV), and found that hand-hygiene practices were associated with a reduced likelihood of infection among health workers, but the authors did not carry out a meta-analysis. Saunders-Hastings and colleagues[32] (2017) also found that regular hand hygiene practices were associated with discernible protective effects against pandemic influenza infection (odds ratio [OR] 0.62; 95% CI 0.52-0.73) during the 2009 pandemic, although this review included studies both from healthcare and non-healthcare settings. All three reviews pointed to the urgent need to generate a higher quality of empirical evidence on the effectiveness of hand hygiene practices on reducing infections among health professionals in the context of health emergencies and viral outbreaks.

In the early days of the COVID-19 emergency, Jefferson and colleagues updated results from their 2011 Cochrane review,[30] with a focus on evidence generated by randomised controlled trials (RCTs) and cluster-RCTs prior to the onset of the current pandemic. This review also included studies that took place outside the context of a

health emergency. The authors found that enhancing hand hygiene yielded protective effects against infection (relative risk [RR], 0.89; 95% CI 0.84-0.95) for respiratory illnesses including acute respiratory illnesses, influenza-like illness and laboratory-confirmed influenza, although nearly all evidence came from non-healthcare settings (e.g., schools, households). They further pointed to the notable variations in effect size, depending on the study context and target population.[30] In a pooled analysis of hand hygiene interventions against respiratory illnesses, the study found that the magnitude of the estimated coefficient of interventions targeting adults was higher (RR, 0.85; 95% CI 0.79-0.92) than the estimated coefficient of those targeting children (RR, 0.92; 95% CI 0.84-1.01), although the confidence intervals overlapped.[30]

A growing strand of literature focused on the determinants of compliance with hand hygiene guidelines among health workers, but very little evidence exists on the degree to which health workers comply with hand hygiene guidelines during health emergencies. In one systematic review, White and colleagues (2020)[33] (2020) concluded that the current evidence is insufficient to shed light on the main determinants of compliance with hand hygiene guidelines in the context of outbreaks. Other literature reviews with a much broader scope suggested that contextual factors may significantly influence the behaviours of health professionals around hand hygiene and affect the degree to which they comply with existing guidelines.[34,35] The contextual factors may span from easy access to cleaning facilities, the availability of auditing and feedback mechanisms, and health provider knowledge. For instance, a 6.3% increase in compliance with hand hygiene guidelines was achieved by a RCT that used a complex intervention comprising increased accessibility to cleaning facilities (e.g.,

alcohol-based gel dispensers outside all patient rooms), while also introducing small seminars, performance-feedback mechanisms and informational materials (e.g., pamphlets and posters) in health facilities at the same time.[36]

Available evidence suggests that hand hygiene interventions with multiple components may achieve greater compliance compared to single-component interventions. For instance, one systematic review and meta-analysis examined the evidence on compliance with hand hygiene guidelines generated by RCTs, cluster-RCTs and quasi-experimental designs.[37] This review concluded that compliance rates were higher for interventions that included multiple components of the WHO "My 5 moments for hand hygiene' approach compared to single interventions. A subsequent Cochrane review found that the available evidence is not sufficient in inpatient care settings to determine which precise combination of the WHO 'My 5 moments' approach yields the highest degree of compliance with hand hygiene practices.[38]

Modelled intervention

In this analysis, we modelled a bundle intervention that aimed to increase the number of health workers who practice hand hygiene and enhance the quality of existing hand hygiene practices compliant with WHO guidelines. As a bundle, the model intervention consisted of several components implemented at the same time. These components closely reflected the design features of hand hygiene interventions recommended by WHO and have been shown effective in curbing infection rates in health professionals, while achieving a high degree of compliance.[39,40]

The first component aims to improve access to cleaning facilities and supplies in healthcare facilities. This entails procuring cleaning supplies and facilities including soap and water, soap dispensers, towels, and alcohol-based handrub.[40] Once procured, these supplies and facilities will be set up within the health facility as recommended by WHO (e.g., at the point of care, near toilets or areas where waste is disposed, public areas, etc.), maintained and stocks regularly replenished.

The second component entails building an educational programme for hand hygiene, with the aim of raising awareness and knowledge and developing core competencies. This component involves identifying one full-time IPC focal point per 250 beds (e.g., nurse or physician) who will be responsible for carrying out hand hygiene training at least once each year. Each training session is assumed to last 2 hours and focus on the importance of hand hygiene in five key moments in the course of care provision.[40] These moments include prior to and after physical contact with any patient, irrespective of the COVID-19 status of that patient; before undertaking a clean or aseptic procedure; following exposure to bodily fluids; and after contact with the immediate surroundings of any patient.[41] Teaching sessions are assumed to be carried out in a dedicated space within the health facility. In each training session, teaching materials will be distributed. Lessons learnt during the sessions will be reinforced by the third component of the modelled hand-hygiene intervention, which entails making information materials (eg, brochures, posters) available in each health facility. To this

end, information materials will be printed and placed in visible spaces within health facilities.

Another component of the modelled intervention consists of putting in place audit and feedback mechanisms to monitor compliance with hand hygiene guidelines and integrate feedback from health professionals. Integrating feedback may be particularly important in the context of the ongoing pandemic as the level of compliance with hand hygiene practices has been suggested to fluctuate in the course of the pandemic.[42] By integrating feedback, course corrections may be introduced to keep compliance rates high over the simulation period. This component will involve identifying a trained IPC focal point who will monitor ongoing hand hygiene practices at the health facility, carry out observational monitoring of relevant units at least once each year, and report findings to relevant stakeholders. The last component entails efforts to foster a professional culture conducive for adopting best practice in hand hygiene and consists of appointing a coordinator who will champion hand hygiene promotion at the health facility. Therefore, the coordinator will spearhead the development and implementation of hand hygiene action plans and produce communication materials to promote a high degree of compliance with hand hygiene guidelines.

Effectiveness estimates

We used the pooled effectiveness estimates generated by Jefferson and colleagues (2020) based on studies focusing on adults, with a relative risk of infection equivalent to 0.85 (95% CI 0.79-0.92).[30] Our choice of the effectiveness estimates for enhancing hand hygiene comes with certain caveats. Studies included in the review by Jefferson

and colleagues took place prior to the onset of the COVID-19 pandemic and most evidence generated in these studies were from outside of health facility settings, with notable variations in the intervention design. Yet, in the context of the ongoing outbreak, it is plausible that the benefits of hand hygiene are more salient to health workers than the general population, given that they are repeatedly exposed to infected patients, especially in health systems experiencing staff shortages. Despite these caveats, we opted to use the estimates provided by Jefferson and colleagues[30] as this review evaluated evidence generated by studies with designs offering a higher quality of evidence (i.e., RCTs and cluster RCTs) compared to other study designs (e.g., observational studies). Given these caveats, we argue that the pooled effectiveness estimates used in this analysis may provide a conservative approach for the effectiveness of hand hygiene interventions in health care settings.

Intervention 2: Increased access to PPE

Overview of the literature

Growing evidence underlines the effectiveness of increasing the use of PPE among health professionals in the context of the ongoing outbreak.[44–45] As an example, Chou and colleagues (2022)[31] recently demonstrated that PPE use was consistently associated with a reduced risk of infection among health workers based on evidence from current and past outbreaks, although most of the relevant evidence came from previous health emergencies, such as SARS-CoV-1 and MERS-CoV. They also pointed out that no studies had examined the effects of PPE re-use on the likelihood of SARS-CoV-2 infection among health workers.

Emerging evidence suggests that the configuration of PPE may have implications for the degree of protection against infection. Chou and colleagues[31] found that masks have a consistent protective effect against infections in healthcare during health emergencies. The authors also found protective effects for other PPE types (e.g., gloves, gowns, eye protection), but evidence was less consistent compared to mask-wearing. Findings from this review are aligned with other systematic reviews that examine evidence between physical interventions to interrupt the spread of infections in healthcare and non-healthcare settings.[30] In their most recent review, Jefferson and colleagues[30] conducted a meta-analysis based on RCTs and cluster RCTs included in their analysis and demonstrated that different configurations of PPE may influence the level of protection against infection. However, to date, no studies have provided empirical evidence on the optimal configuration of PPE that may achieve the greatest reductions in the risk of infection among health workers in the context of the ongoing COVID-19 outbreak.

The beneficial effects of efforts to increase the availability of PPE may be bolstered by providing PPE education. Similar to other studies, one recent Cochrane review by Verbeek and colleagues (2020)[46] found that the use of full-body PPE was linked with greater protection against highly infectious diseases. The authors also found that PPE education could lower the likelihood of errors in the use of PPE (e.g., fewer mistakes in doffing PPE). They further suggested that the choice of training methodology may influence the level of compliance with the existing PPE guidelines, with face-to-face PPE training sessions offering a more effective strategy for reducing non-compliance with existing PPE guidance compared to video-based teaching sessions.[46]

Modelled intervention

The modelled intervention aimed to improve the availability of PPE in line with WHO guidelines through two components. The first component entailed building a real-time PPE tracking system to take stock of different types of PPE available at health facilities. This component recognises that keeping track of PPE needs in the context of a prolonged and evolving health emergency can be daunting, particularly in resource-constrained settings. It involves identifying an on-site managerial staff member who is already tasked with addressing occupational safety concerns at the health facility. The on-site manager is assumed to oversee the coordination with local/national health authorities through the PPE tracking system to ensure a steady supply of PPE at the facility. It is also assumed that this staff member will calculate the PPE use rate using a standard methodology, similar to the free-of-charge, Excel-based calculator developed by the United States Centers for Disease Control and Prevention, and thus ensure that all health workers are aware of PPE available at the facility.[47]

The second component relates to the procurement of PPE according to the latest WHO guidelines. This includes procuring medical masks, disposable fluid-resistant gowns, gloves, and eye protection (e.g., face-shields, visors, goggles), scrubs, disposable aprons, safety boxes for needles/syringes, disposable bags for biohazard waste, particulate respirators, and fit test kits to ascertain the effectiveness of seal for tight-fitting respiratory protection devices.[48] In addition, PPE used during aerosol-generating procedures, e.g., tracheal intubation, non-invasive ventilation, tracheotomy, cardiopulmonary resuscitation, manual ventilation before intubation, bronchoscopy, sputum induction using nebulized hypertonic saline, and dentistry and autopsy

procedures, should also be procured.[48] In line with WHO guidelines, each PPE should be considered for single-use only and should not be worn repeatedly while providing care to different patients.

Effectiveness of intervention

In our study, we calculated that individuals who have access to PPE are about 64% less likely to get infected compared to those with no access, with an estimated relative risk of SARS-CoV-2 infection equivalent to 0.36 (95% CI 0.0.24-0.533). We derived this estimate through a two-step approach. First, we extracted the estimates on the effectiveness of PPE against the risk of infections from the meta-analysis carried out by Chu and colleagues and reviewed the characteristics of studies included in this analysis.[45] Chu and colleagues estimated that the use of masks can help lower the risk of infection in healthcare settings with a relative risk equivalent to 0.34 (95% CI 0.24-0.47). Similarly, the use of eye protection (e.g., goggles and eye shields) was associated with reductions in the risk of infection, with a relative risk of 0.33 (95% CI 0.20-0.56).[45] We relied on an additive combination of these estimates for masks and eye protection to derive the effectiveness of PPE against SARS-CoV-2 infections, which yielded a relative risk estimate of 0.11 (95% CI 0.08-0.15). We assumed that the protective effects of the PPE would not vary over time.

We note that the review of Chu and colleagues included studies of any design across all types of settings that included an analysis of patients with a confirmed/probable diagnosis of infection with SARS-CoV-2, SARS-CoV or MERS-CoV according to WHO guidelines, as well as people who were in close contact with these patients.[31] The authors concluded that the evidence included in their study was marked by a low level of certainty, which may impact on the precision of point estimates. In our study, we

opted to use evidence from Chu and colleagues as this study included evidence generated through studies in the context of the COVID-19 pandemic, as well as previous outbreaks, and used statistical analysis methods, such as Bayesian meta-analyses, to derive effectiveness estimates and inform the interpretation of results.

Our review of studies included in the meta-analysis of Chu and colleagues and relevant to access to PPE suggested that they generally included interventions that combined increased access to PPE with IPC education and training,[31] which prompted us to remove the effectiveness of IPC education and training. To do this, we examined the results generated by Chou and colleagues.[31] In their review, the authors identified studies that included evidence on the potential effectiveness of IPC training and education interventions in reducing the risk of infection among health workers in the context of health emergencies, including SARS-CoV-2, SARS-CoV-1 and MERS-CoV.[31] We performed a meta-analysis based on studies included in their review that provided sufficient information (see Table A.7. for the list of studies).[31] Our analysis suggested that providing IPC training and education for health workers may result in a reduction in the risk of infection among this population (RR, 0.31; 95% CI 0.24-0.39).

Table A.7. Studies used in the calculations for the individual effectiveness of IPC education and training interventions.

Author	Year of publication	Country	Setting	Outbreak	IPC training
Chen et al.	2009	China	Hospital	SARS-CoV-1	Specialised training on SARS-CoV-1
Lau et al.	2004	Hong Kong (SAR), China	Hospital	SARS-CoV-1	Specialised training on SARS infection control
Liu et al.	2009	China	Hospital	SARS-CoV-1	Training on infection control protocols
Pei et al.	2006	China	Hospital	SARS-CoV-1	Specialised training on SARS prevention
Raboud et al.	2010	Canada	Hospital	SARS-CoV-1	SARS-specific infection control training

The confidence intervals for the effectiveness estimates associated with this intervention were computed using a bootstrap approach. The effectiveness of each intervention is sampled 1000 times using a PERT distribution (parametrised using the mid, min, max of the single intervention's effectiveness). These samples are then combined using an additive in the principal analysis or multiplicative approach as a sensitivity check to obtain 1000 samples of the combined effectiveness of the intervention. In the next step, mid-estimates and the 95% CIs of the combined intervention are deduced from those samples.

Intervention 3: Combined increased access to PPE and IPC education and training

Overview of the literature

Increasing the knowledge of IPC practices may not be sufficient on its own if health workers do not have adequate access to PPE designed with the aim of minimizing

exposure to hazards and/or are unable to appropriately use the existing PPE. In recognition of this issue, the modelled third intervention combined increased access to PPE with IPC education and training.

To our knowledge, only a few studies have investigated the relationship between IPC training and the risk of SARS-CoV-2 infections among health workers. One survey conducted with asymptomatic health workers in Italy found that the positivity rate was substantially higher among those that did not attend IPC training sessions on PPE use compared to those that were in attendance (OR 2.86; 95% CI 0.14-56.58).[49] Similarly, in a cross-sectional survey with more than 3000 health workers, Zhou and colleagues (2020) found that the positivity rate among infected health workers that attended a COVID-19 knowledge training was approximately one-third of the positivity rate among those that did not participate.[50]

Although limited evidence from previous outbreaks points to the protective effects of IPC education and training, Chou and colleagues found evidence suggesting that IPC training was consistently associated with reductions in the risk of infection among health workers in the context of SARS-CoV-1 and MERS-CoV outbreaks.[31] In addition, IPC training and education programmes have been shown to increase provider knowledge of IPC practices, improve provider competency, and increase compliance with existing guidelines by 27.5%.[51,52] A growing body of evidence shows that simulation-based IPC training is associated with a reduction in healthcare-associated infections,[52] including central line-associated bloodstream infections.[53,54]

Modelled intervention

In addition to the components of the PPE only intervention, the modelled intervention also consisted of a mandatory IPC educational programme, with the aim of standardising IPC practices in healthcare settings. In each health facility, one trained health worker will take the additional responsibility as an IPC focal point with dedicated time per 250 beds as per WHO guidelines.[40] In this capacity, the IPC focal point will coordinate all IPC educational activities (e.g., curriculum development) and practical matters (e.g., booking a physical space within the health facility allocated for each training session). S/he will be selected from staff members with sufficient time and an appropriate skillset to ensure that IPC training sessions are provided with no disruptions, and also coordinate with academic institutions and relevant local stakeholders to ensure that teaching materials are contextually appropriate and kept up-to-date with the evolving guidance for best IPC practices.[40]

IPC training sessions will last 2 hours and be carried out at least once each year. Each training session will take place within the health facility. During each session, handouts will be distributed to all participants. Teaching materials will closely reflect the open-access IPC training course developed by WHO specifically with the ongoing pandemic in mind. IPC learning outcomes will reflect theoretical knowledge and practical IPC skills. This includes covering the underlying rationale for IPC interventions and explaining how each IPC intervention can interrupt the transmission of SARS-CoV-2 infection. Sessions will also focus on practical considerations (i.e., respiratory etiquette, waste disposal) according to the latest guidelines by WHO and others[55] and encourage

creating a professional environment that fosters a high degree of compliance with IPC guidelines. Teaching methods will emphasize team- and task-based strategies. Sessions will encourage participation and involve simulation training in line with existing evidence that these teaching methods are associated with reductions in other healthcare-associated infections.[54,56] Evidence demonstrates that access to accurate information at the point of service is crucial to encourage best IPC practices.[35] In recognition, IPC guidelines will be made available to all health workers. After completion of each teaching session, the IPC focal point will collect feedback from health workers who participated in the session, report this feedback to relevant stakeholders, and modify the course content as needed. Information materials (e.g., posters and brochures) will be printed and made available in visible physical spaces within facilities as a reminder of best IPC practices.

Effectiveness estimates

In our study, we estimated that individuals who have access to PPE and IPC education and training are about 88.8% less likely to get infected with SARS-CoV-2 compared to those who do not have access to this intervention, with the relative risk equivalent to 0.11 (95% CI 0.08-0.15). As before, we derived this estimate using the effectiveness of PPE as an additive of estimates for masks and eye protection extracted from Chu and colleagues.[44] We computed the confidence intervals for this intervention using the same approach described in the earlier intervention focusing on increased access to PPE.

Establishing baseline coverage of the modelled IPC interventions

To identify baseline intervention coverage, we first identified the 2019 WHO IPC Self-Assessment Framework (IPCAF) survey questions that are most relevant to a given intervention.[25] Baseline coverage of the intervention to promote best practices in hand hygiene was assessed on questions related to whether reliable and functional hand hygiene stations (e.g., alcohol-based handrub solution, etc.) were available; whether the health facility had hand hygiene guidelines; availability of staff with IPC expertise to lead IPC training; and whether all health workers received at least one mandatory IPC training session each year. Baseline coverage of the modelled intervention on scaling-up PPE availability was assessed on whether PPE and disposable items were available at all times and in sufficient quantity for all health workers. This question was also used to determine the baseline coverage of the intervention that combined increased access to PPE and IPC education and training, in addition to questions that examined whether staff with IPC expertise to lead IPC training were available, and whether all health workers received at least one mandatory IPC training each year. We then calculated the sum of IPCAF scores of each health facility for each modelled intervention separately, which can be interpreted as whether the health facility had the IPC capacity at baseline in congruence with the modelled IPC intervention. Regional averages were then calculated by aggregating facility-level IPCAF scores, adjusting for the population size of each country.

While determining the baseline and target coverage, we assumed that there was a one-to-one relationship between the percentage of health facilities with the required IPC capacity in line with the modelled IPC interventions and the percentage of health

workers to whom these interventions will reach. In other words, if 50% of health facilities in a geographic area are assumed to have IPC capacity in alignment with the modelled IPC intervention at baseline, this is interpreted as 50% of all the health facilities and health workers in that area are assumed to be covered by that intervention. Table A.8. depicts the percentage of health facilities in each region that had the IPC capacity in line with the modelled IPC interventions. For instance, it is estimated that 56% of health facilities in OECD countries had the IPC capacity to implement the modelled IPC intervention on promoting hand hygiene compared to 15% of health facilities in non-OECD countries in the Americas region.

Table A.8. Percentage of health facilities that align with the modelled IPC interventions at baseline by WHO-OECD regions/countries (population-weighted). OECD calculations based on IPCAF survey.

**Modelled intervention**	**Promoting best practices in hand hygiene**	**Scaling-up access to PPE**	**Combining increased access to PPE and IPC education and training**
OECD countries	56%	51%	37%
non-OECD countries in the Americas region	15%	16%	7%
non-OECD European region countries	23%	29%	10%
non-OECD Western Pacific region countries	49%	47%	28%
Eastern Mediterranean region countries	33%	42%	24%
African region countries	7%	10%	4%
South-East Asia region countries	15%	16%	9%

Calculating intervention costs

Total costs of interventions were calculated as the sum of two budget lines: 1) programme-level costs, which include administration, training and other activities taking place above the health worker level; and 2) expenditures at the health worker-level, which include the personal use of goods and services such as PPE. A standardised, ingredient-based approach was used to calculate expenditures for both cost lines. The approach required information about the quantities of physical inputs needed and their respective unit cost. Where prices and inputs varied (e.g., due to the country-specific context), we attempted to use country-specific estimates. Conversely, the same inputs and costs were used across countries when this was considered a credible assumption. For example, the same price was used across countries for goods that are traded internationally, such as PPE. Similarly, the quantity of PPE for a given type of health worker or the amount of training provided were maintained as a constant across countries given that the quantity of these inputs was based on international guidelines.

Multiple sources of data were used to identify both the inputs and the unit price costs. The key data to model inputs and costs for programme-level costs were derived from the WHO-CHOICE database, as well as from previous OECD publications on infectious diseases that focused on promoting hand hygiene and enhanced environmental hygiene in healthcare services.[26,57] Information on health worker-level inputs and prices were primarily based on the essential supplies forecasting tool (ESFT), developed by the Clinton Health Access Initiative to forecast the needed supplies for an effective clinical response during the COVID-19 pandemic.[58]

Programme costs at the regional level were calculated in two steps. First, average costs per inhabitant were calculated at the national level for a set of countries whose combined population size would be at least 90% of the total population in the region. This simplification allowed to decrease the computational time of the analyses, while maintaining a high degree of reliability. In a second step, the average regional costs per person were calculated by averaging the country-level estimates, while accounting for the population size of each country. Thus, a higher weight was given to more populous countries in the region. The region-specific average programme cost per inhabitant was then multiplied for the population size of the region, derived from the OECD SPHeP-COVID model, for the calculation of the total programme-level costs.

Total expenditure at the health worker-level mainly consisted of costs for commodities for personal use. It depends on the number of people hospitalised and admitted to ICUs as calculated by the OECD SPHeP-COVID model and the assumptions on the use of commodities from the ESFT. Patients in ICUs require health workers adhering to more stringent protocols, which result in a higher use of PPE and more frequent hand hygiene. The use of PPE and products for hand hygiene practices are thus lower for health workers taking care of patients in hospitals, but not admitted to ICUs. The quantity of inputs per patient per day in the hospital or ICU, as well as the cost per item, were considered to be constant across geographical settings.

The costs of the intervention to enhance hand hygiene included the establishment or the strengthening of a central coordination mechanism to support the implementation of the intervention across healthcare services in the country. The coordination mechanism

was established both at the national and subnational level under the assumption that this was a relatively simple public health programme, which would remain stable throughout the implementation period. The coordination mechanism involved both administration, supervision and monitoring, as well as training and evaluation, both at the national and subnational level. The intervention also entailed the purchasing of supplies to allow hand hygiene practices following guidelines, e.g., sufficient supplies of alcohol-based handrub or liquid soap.

The cost of scaling-up access to PPE included both programme- and personal-level costs. Similarly, to the enhancing hand hygiene intervention, programme-level costs included the establishment or strengthening of an administrative institution in charge of management, supervision and monitoring of the intervention. The programme was considered to be more complex than the one to enhance hand hygiene, mainly because it was assumed that the number of inputs is higher and activities are more elaborate. The intervention also entailed the purchasing of supplies such as scrubs, masks, face shields, gowns, and goggles. When combined with an IPC education and training component, the intervention also entailed the rolling-out of a programme based on a comprehensive approach. The main cost items of the approach included: 2-hour training sessions provided to groups of health workers at the health facility-level; provision of guidelines and visual reminders; and provision of feedback. The costs, expressed in 2020 US$ at PPP should be considered as additional to those existing before the COVID-19 pandemic, based on data from the 2019 WHO IPCAF.[25] Only costs borne by the healthcare sector were considered.

Based on the methodology and the assumptions mentioned, the resulting total cost per

person by region were those presented in Table A.9. Programme-level costs ranged between 55% and 99% of total costs, with the remaining share devoted to purchasing commodities. Programme-level costs tended to account for a smaller share of total costs in interventions requiring a higher use of commodities and consumables, such as scaling-up access to PPE. In terms of the geographical setting, programme-level costs tended to account for a larger share of total costs in lower-income regions. To a large extent, this can be explained by two factors. First, coverage of IPC interventions was lower in these settings in the baseline scenario, which then resulted in a higher quantity of inputs needed in the scenario intervention to meet the target. Second, commodities and consumables traded on the international market have a higher relative cost in these countries compared to other country-specific inputs than in countries with a higher income level.

Table A.9. Intervention costs and cost composition by region (2020 US$ PPP per capita)

	**Enhancing hand hygiene**		**Scaling-up access to PPE**		**Increased access to PPE and IPC education and training**	
	Total cost per capita	Programme cost (% of total costs)	Total cost per capita	Programme cost (% of total costs)	Total cost per capita	Programme cost (% of total costs)
OECD countries	0.52	99%	1.24	91%	2.80	94%
African region countries	0.30	96%	0.68	64%	0.69	62%
Non-OECD countries in the Americas region	0.23	95%	0.73	67%	1.68	84%
Eastern Mediterranean region countries	0.31	98%	0.56	83%	0.82	83%
Non-OECD European	0.44	97%	0.89	73%	2.56	88%

region countries						
South-East Asia region countries	0.15	95%	0.38	56%	0.47	61%
Non-OECD Western Pacific region countries	0.14	96%	0.34	55%	0.64	63%

Sensitivity analysis: pandemic scenarios using different reproduction numbers

We assessed the sensitivity of our results on the effectiveness of the modelled IPC interventions in reducing infections under different scenarios for the reproductive number. To do this, we performed our principal analyses, but this time the R_o ranged from 1.5 to 5. As shown in Fig. A.3., our results suggested an inverse relationship between the effectiveness of the three modelled interventions and the reproductive number. In all regions, the effectiveness of the hand hygiene intervention diminished as R_o increased, and the beneficial effects virtually disappeared in most regions when R_o reached the highest value. Similarly, the effectiveness of PPE only and PPE+ interventions diminished with R_o in most regions, with a variation of the magnitude of the declining effectiveness across regions. For both PPE only and PPE+, the largest declines in the effectiveness of these interventions were observed in the Africa and South-East Asia regions, and the smallest among OECD countries and non-OECD countries in the Western Pacific region. This finding suggested that the diminishing protective effects of PPE only and PPE+ interventions were particularly pronounced in settings where the relative risk of infection at baseline was substantially higher among health workers compared to the general community. Our findings were in agreement with earlier studies

that examined the effectiveness of different public health measures under different reproduction number scenarios.[59,60]

New variants of SARS-CoV-2 infection have been in circulation in different settings.[61] Some of the new variants have been suggested to have higher infectivity rates[62] and thus a greater likelihood of resulting in new infections, hospitalisations and mortality.[63] If the infectiousness was greater in the new variants, this would be reflected as a higher reproduction rate. Our results suggested that a higher reproduction number in turn will likely render the IPC interventions less effective in preventing adverse health events among health workers. Taken together, evidence from our study suggested that the emergence of variants with higher transmissibility underscores the pressing need for scaling-up efforts to increase access to PPE and IPC training.

Fig. A.3. Effectiveness of the modelled IPC interventions in reducing infections under different scenarios for Ro. Red dashed line indicates the basic reproduction number (Ro=2.5) used in the main analysis. Vertical axes are not on the same scale.

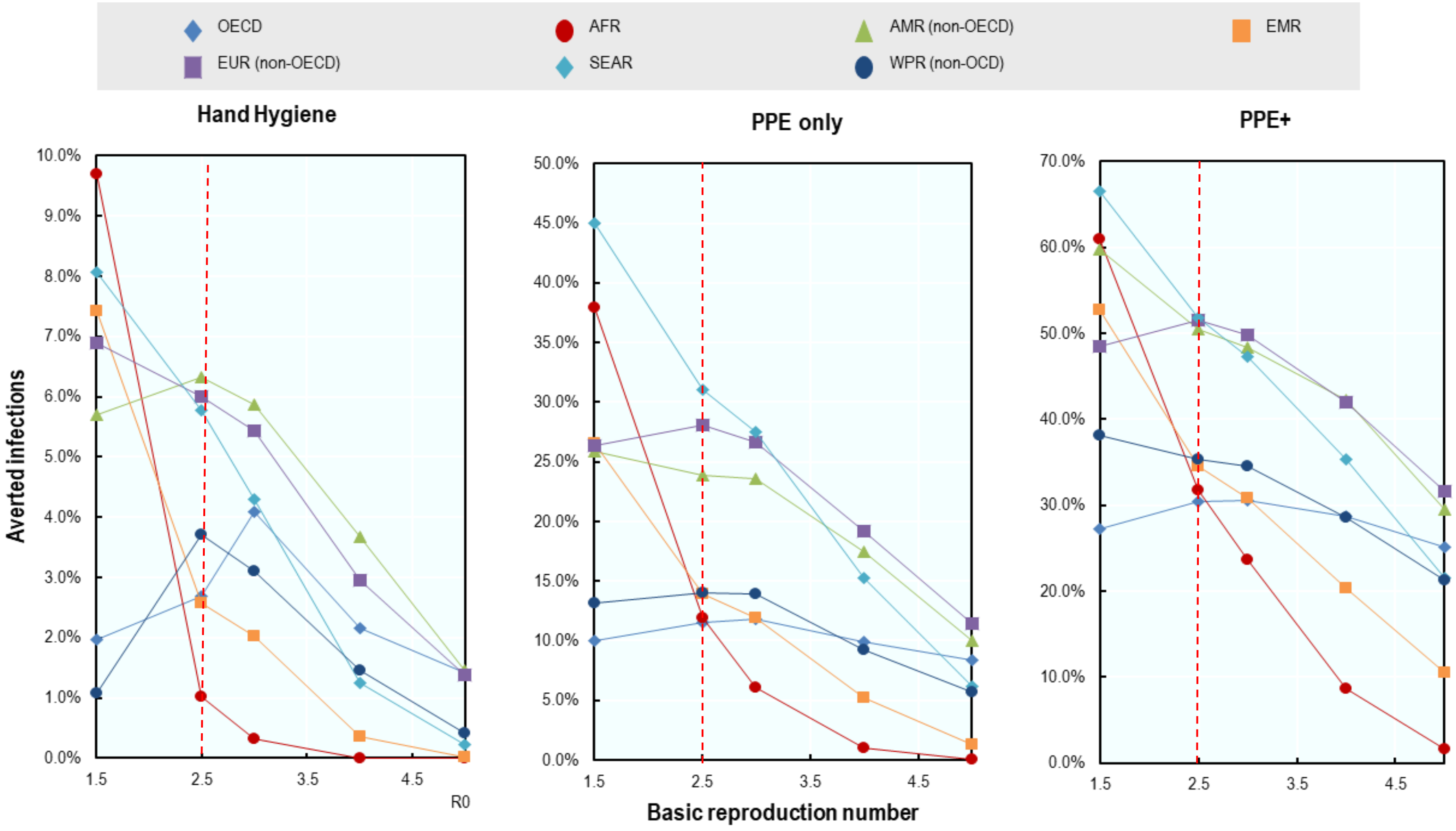

Sensitivity analysis: unmitigated pandemic

The sensitivity analyses depicted below demonstrates how our estimates changed in the case of an unmitigated pandemic scenario where no social distancing measures are implemented at the societal level. As shown in Fig. A.4., our results suggested that only the PPE+ intervention among the modelled interventions could have averted new infections across all regions, although the magnitude of averted infections was lower than in our principal analysis (see Fig. A.5. for the estimated results on averted infections per 100 000 health workers using the unmitigated pandemic scenario, and Fig. A.6. for the estimated costs and savings associated with scaling-up the modelled IPC interventions using the unmitigated pandemic scenario). None of the modelled interventions was associated with statistically significant declines in the number of hospitalisations, ICU admissions and deaths that could have been averted. This finding suggested that our results were sensitive to the societal level, social distancing precautions that influenced the protection of health workers outside of healthcare settings. Similar to earlier studies,[59] our findings suggested that in a scenario where insufficient societal-level mitigation policies are implemented, scaling-up IPC capacity is not completely sufficient on its own to limit the transmission risk among health workers. This is not surprising as health workers face the risk of SARS-CoV-2 infections not only in healthcare settings, but also in their exposure to infected individuals in the community. Evidence generated by our study suggested that policymakers would benefit from prioritising IPC interventions in health facilities, while considering social distancing measures that best fit their own priorities to protect the health of their health workers.

Fig. A.4. Averted cases, hospitalisations, ICU admissions and deaths among health workers attributable to modelled IPC interventions, unmitigated pandemic, by regions. Dark blue represents enhancing hand hygiene (HH); grey represents increasing access to PPE (PPE only); purple represents increasing access to PPE in combination with IPC training and education (PPE+). Error bars represent the 95% confidence intervals for each region.

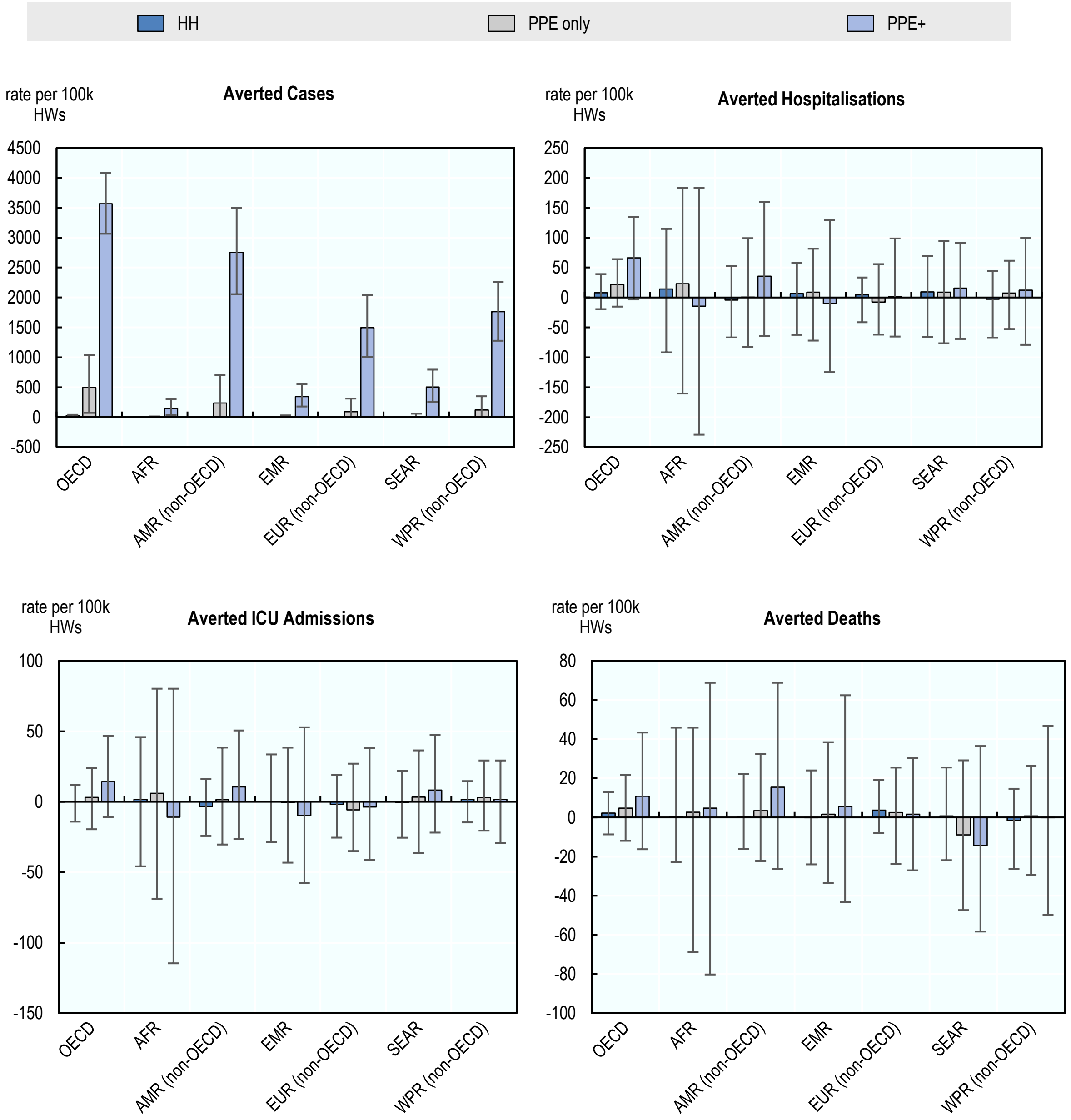

Fig. A.5. **Disability-adjusted life years averted by 100 000 health workers, unmitigated pandemic, by regions.** Modelled IPC interventions are enhancing hand hygiene (HH), increasing access to PPE (PPE only) and increasing access to PPE in combination with IPC training and education (PPE+). Dark blue represents years lived with disability; grey represents years of life lost. Error bars represent the 95% confidence intervals for each region.

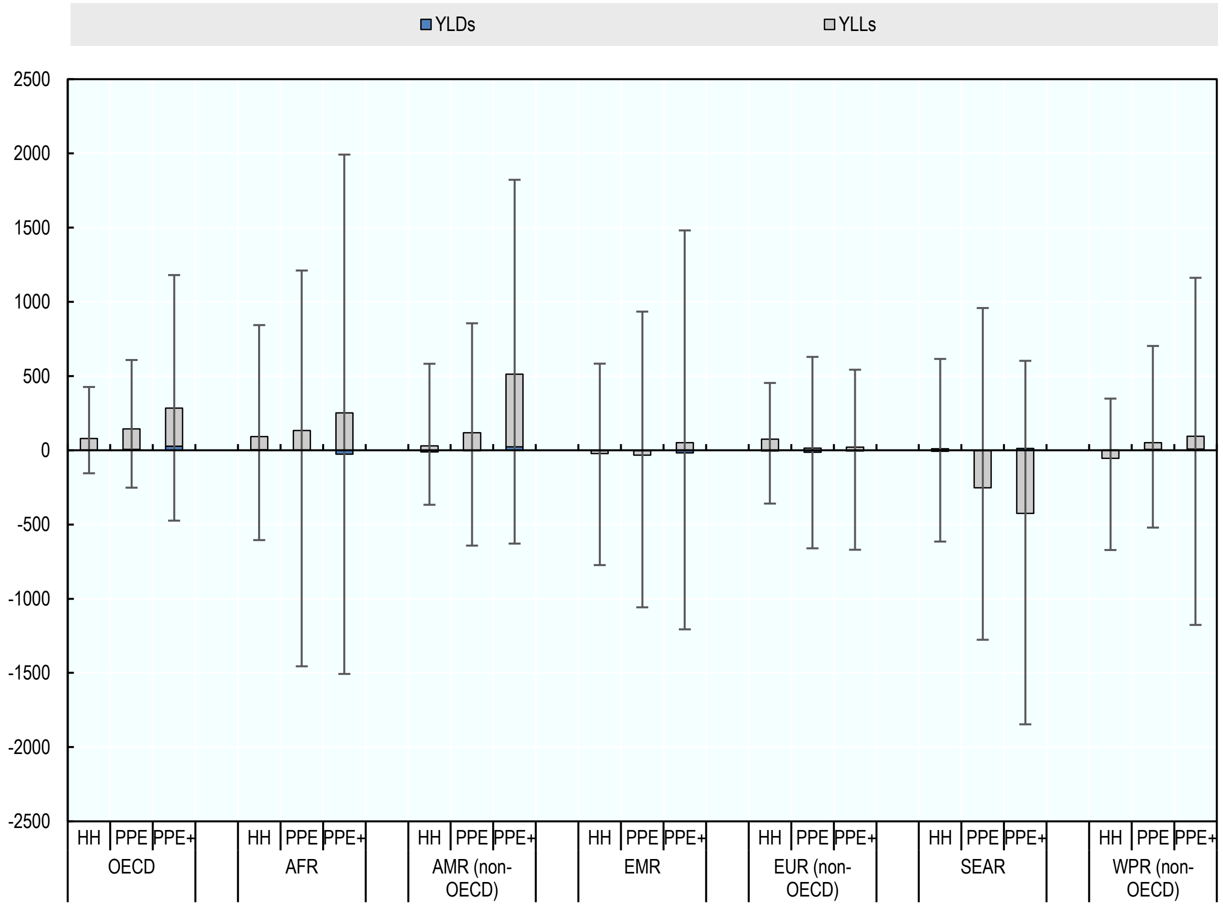

Fig. A.6. Costs and savings associated with scaling-up the modelled IPC interventions, unmitigated pandemic, by regions. Modelled IPC interventions are enhancing hand hygiene (HH), increasing access to PPE (PPE only) and increasing access to PPE in combination with IPC training and education (PPE+). Purple represents health expenditure; grey represents absenteeism; light blue represents premature mortality and dark blue represents total cost. Error bars represent the 95% confidence intervals showing the uncertainty associated with total savings and total costs. Savings are expressed in positive US dollars values and costs are expressed in negative US dollar values.

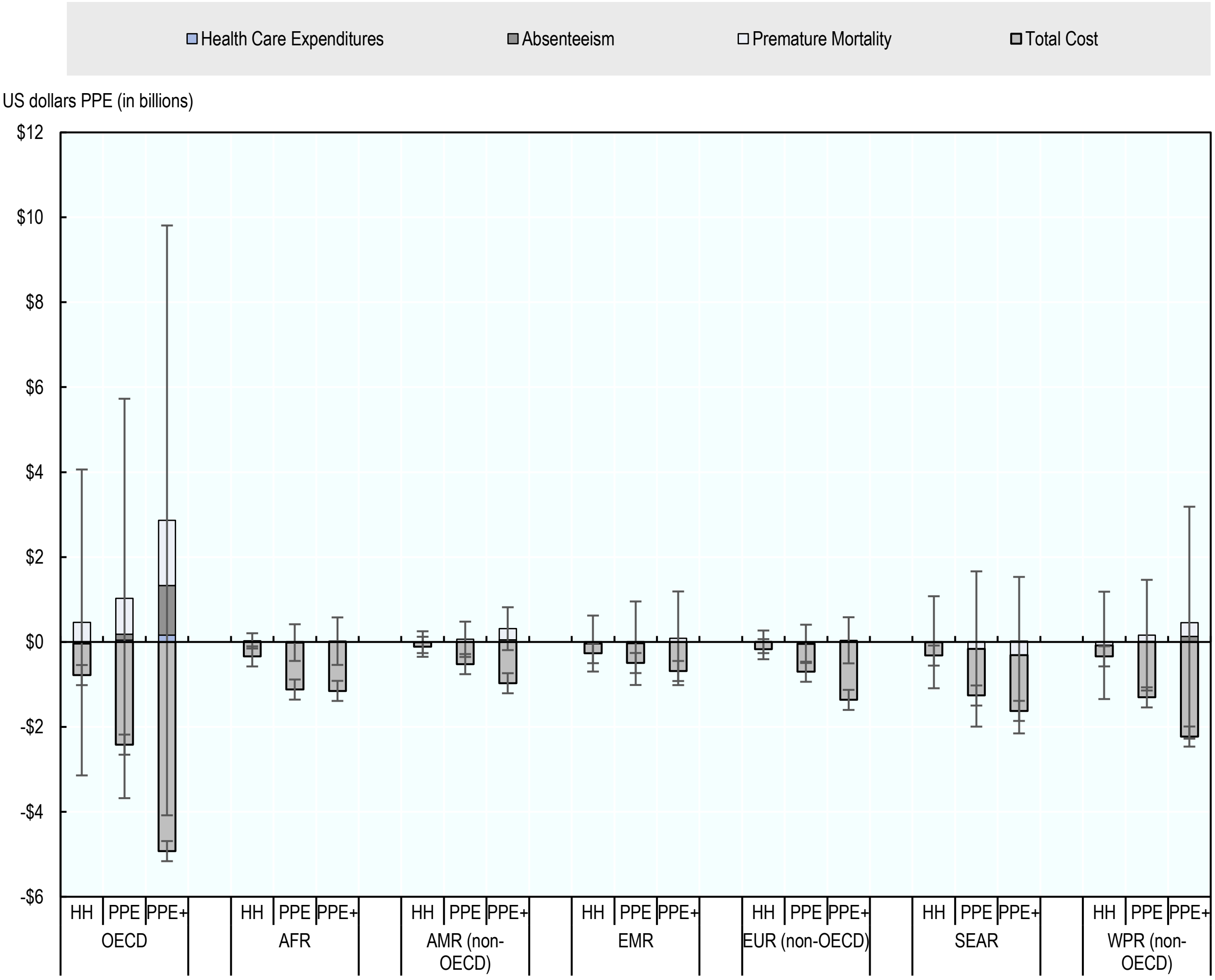

Sensitivity analysis: using multiplicative estimates on intervention effectiveness

The effectiveness of the PPE only and PPE+ interventions can be derived by either an additive approach or a multiplicative approach. Additive approach does not take into account the potential interactions between various components of an intervention (e.g.,

potential interactions of increased access to eye protection and face shields at the same time), whereas the multiplicative approach considers them. In our principal analysis, we presented results on the effectiveness and cost-effectiveness of the PPE only and PPE+ interventions calculated using an additive approach. We opted for the additive approach, because they provided more conservative estimates. As a sensitivity check, we examined the sensitivity of our results by using a multiplicative approach, which yielded relative risk estimates equivalent to 0.17 (95% CI 0.15-0.20) for the PPE only intervention, and 0.37 (95% CI 0.26-0.67) for the PPE+ intervention. As shown in Fig. A.7., Fig. A.8. and Fig.A.9., our substantive results remained robust, although the magnitude of our estimates slightly changed.

Fig. A.7. Averted cases, hospitalisations, ICU admissions and deaths among health workers attributable to modelled IPC interventions using a multiplicative approach, by regions. Dark blue represents enhancing hand hygiene (HH); grey represents increasing access to PPE (PPE only); purple represents increasing access to PPE in combination with IPC training and education (PPE+). Error bars represent the 95% confidence intervals for each region.

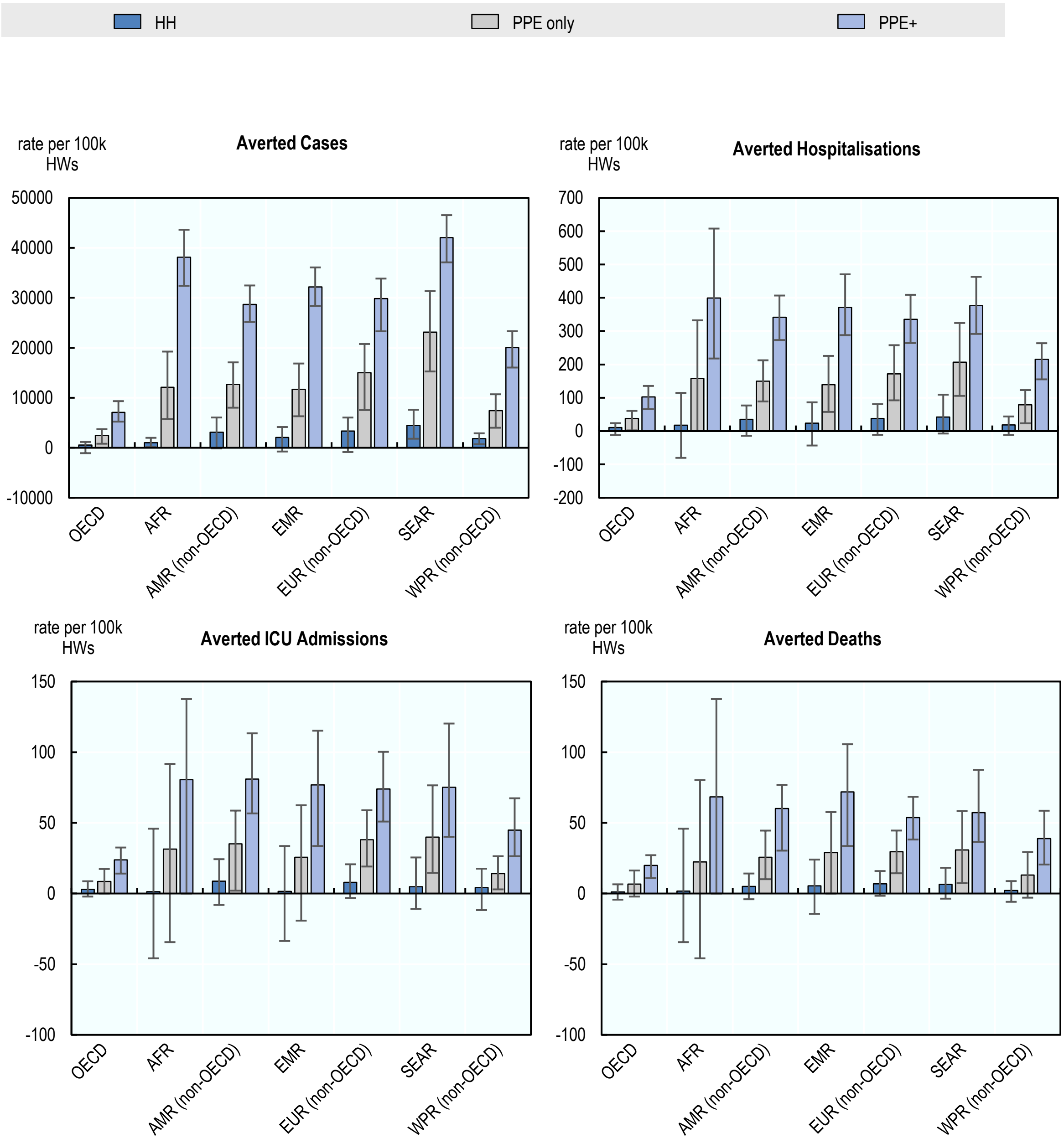

Fig. A.8. Disability-adjusted life years averted by 100 000 health workers using a multiplicative approach, by regions. Modelled IPC interventions are enhancing hand hygiene (HH), increasing access to PPE (PPE only) and increasing access to PPE in combination with IPC training and education (PPE+). Dark blue represents years lived with disability; grey represents years of life lost. Error bars represent the 95% confidence intervals for each region.

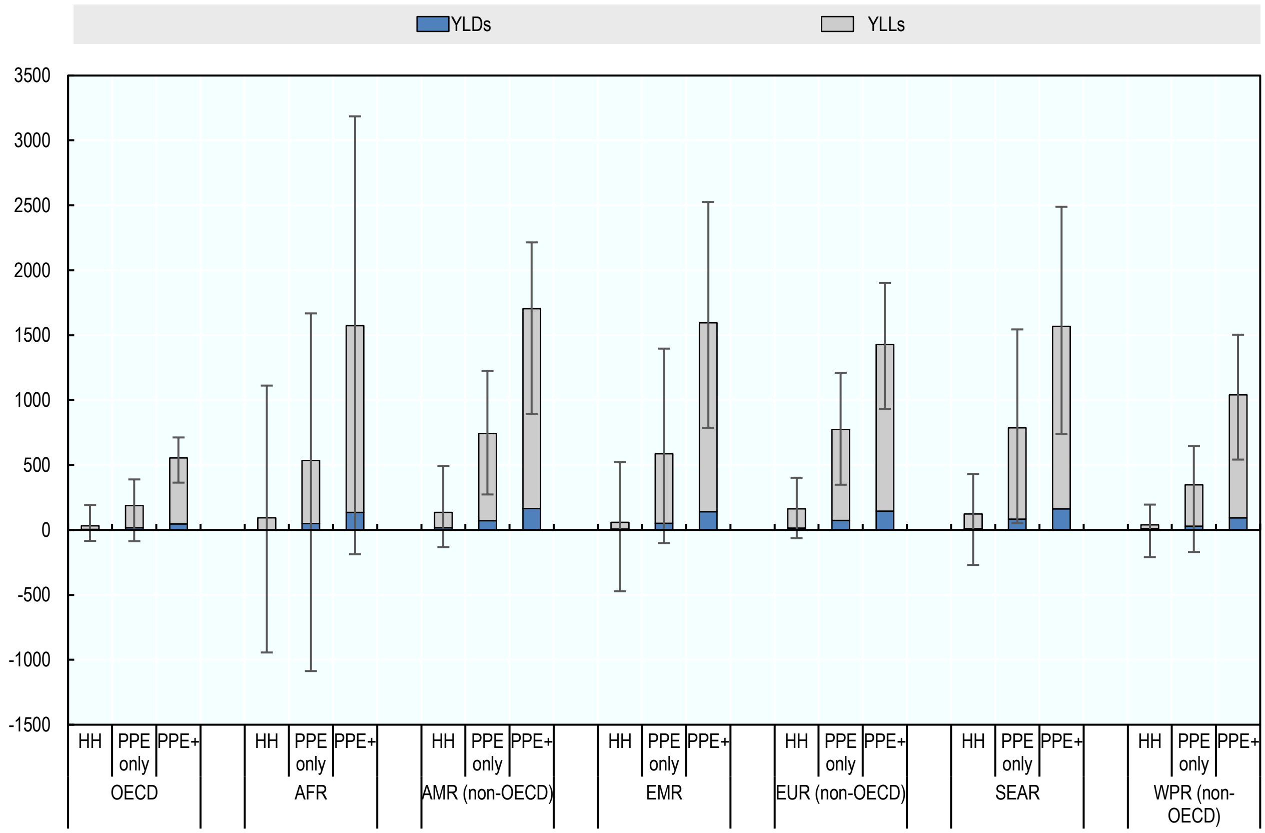

Fig. A.1. Costs and savings associated with scaling-up the modelled IPC interventions using a multiplicative approach, by regions. Modelled IPC interventions are enhancing hand hygiene (HH), increasing access to PPE (PPE only) and increasing access to PPE in combination with IPC training and education (PPE+). Purple represents health expenditure; grey represents absenteeism; light blue represents premature mortality and dark blue represents total cost. Error bars represent the 95% confidence intervals showing the uncertainty associated with total savings and total costs. Savings are expressed in positive US dollars values and costs are expressed in negative US dollar values.

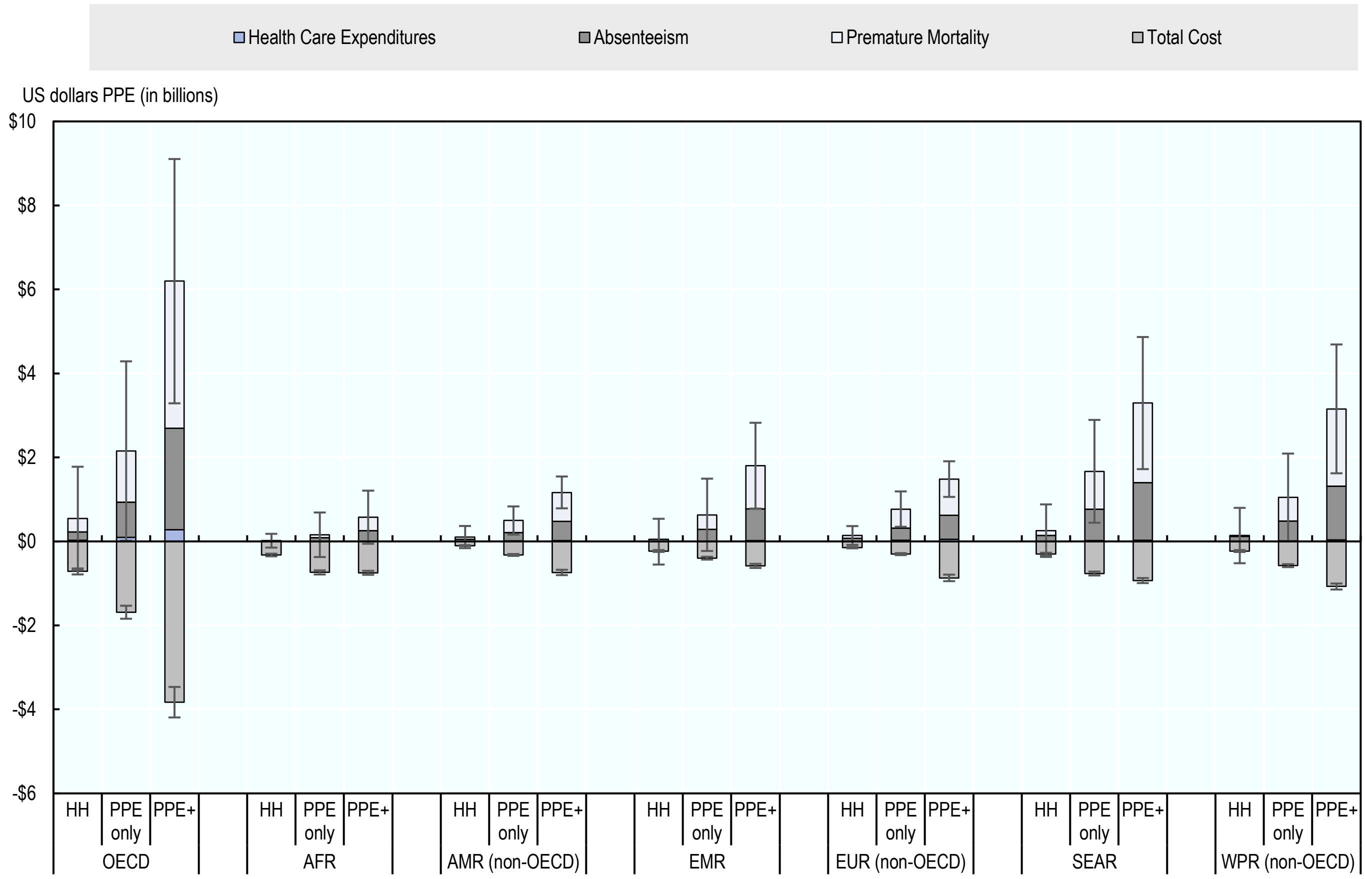

Sensitivity analysis: pro-rating intervention costs

In our principal analysis, the costs covered the first six months of the pandemic, even though implementation of the modelled interventions were often budgeted at the start of each year. As such, our principal estimates presented a conservative approach to quantifying the return on investment of the modelled interventions. As a sensitivity check, we re-ran our principal analysis, but this time pro-rated costs associated with programme-level expenditures (e.g., fixed expenses associated with administrative tasks) to take into account the six-month study period. As shown in Fig. A.10., our substantive results remained robust, although the estimated net savings were greater in magnitude compared to our principal analysis.

Fig. A.10. Costs and savings associated with scaling-up the modelled IPC interventions, pro-rated costs, by regions. Modelled IPC interventions are enhancing hand hygiene (HH), increasing access to PPE (PPE only) and increasing access to PPE in combination with IPC training and education (PPE+). Purple represents health expenditure; dark grey represents absenteeism; light grey represents premature mortality and medium grey represents total pro-rated cost. Error bars represent the 95% confidence intervals showing the uncertainty associated with total savings and total pro-rated costs. Savings are expressed in positive US dollars values and costs are expressed in negative US dollar values.

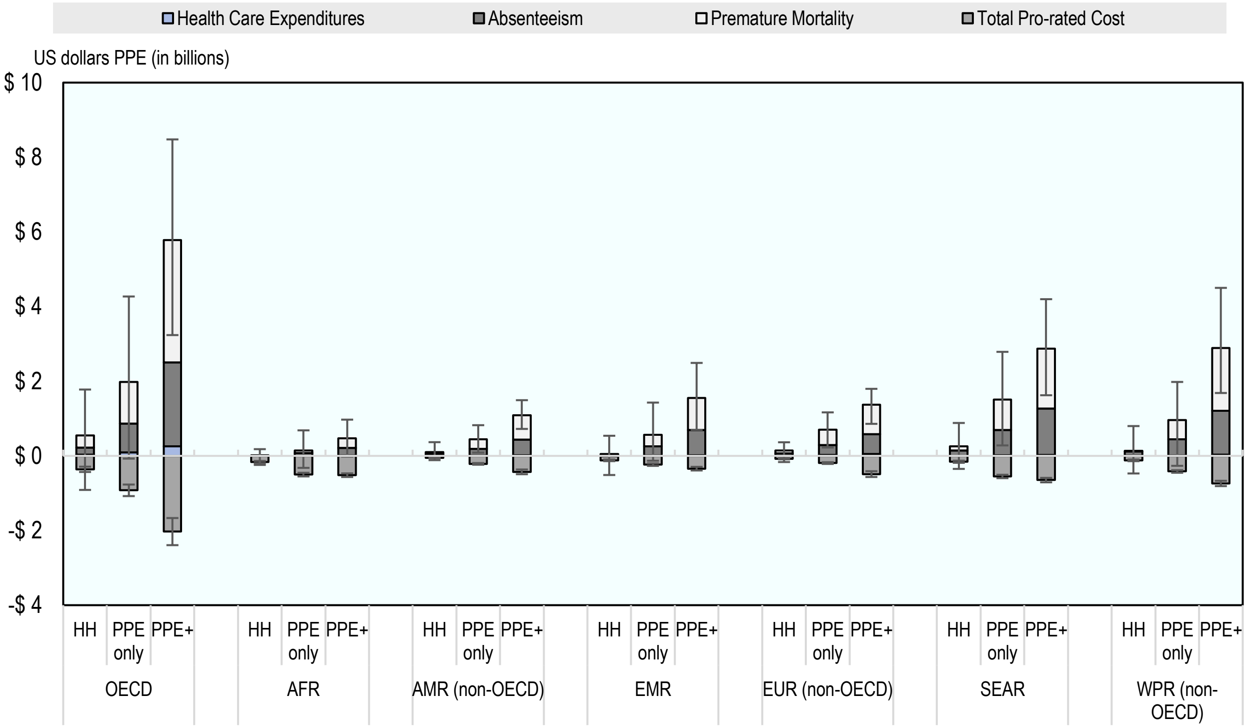

Additional analysis: 100% target coverage

In our principal analysis, we set the target coverage of the three modelled IPC interventions at 80% based on WHO-CHOICE guidelines.[26] Yet, in the context of a pandemic, it is desirable for countries to achieve universal coverage of the modelled IPC interventions so that all health workers can benefit from the protective effects of enhanced hand hygiene programmes, PPE and IPC education and training. With this

consideration in mind, we re-ran our principal analysis and re-set target coverage at 100% (Fig. A.11., Fig A.12., A.13.).

Fig. A.11. Averted cases, hospitalisations, ICU admissions and deaths per 100 000 health workers attributable to modelled IPC interventions, 100% target coverage, by regions. Dark blue represents enhancing hand hygiene (HH); grey represents increasing access to PPE (PPE only); purple represents increasing access to PPE in combination with IPC training and education (PPE+). Error bars represent the 95% confidence intervals for each region.

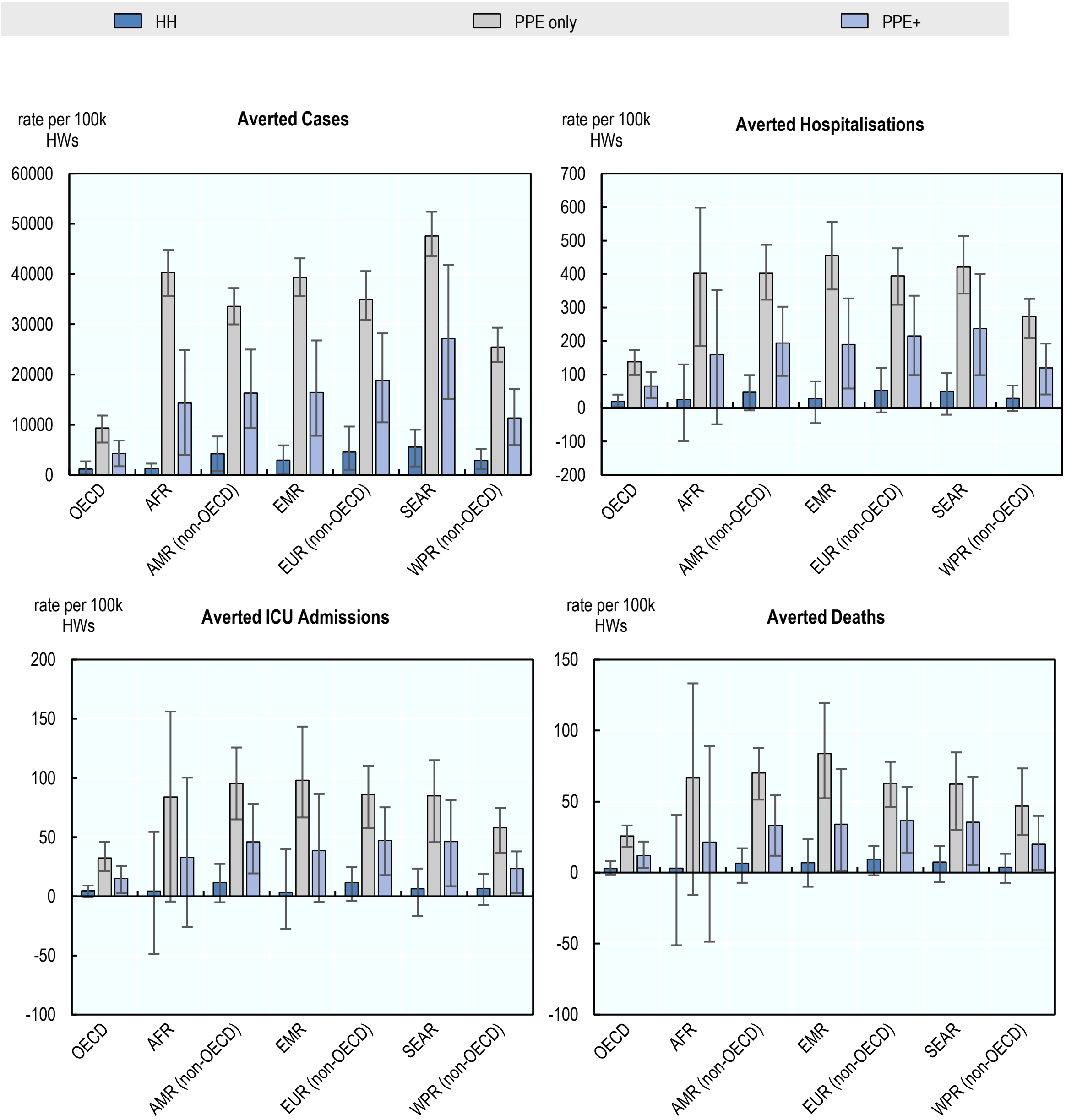

Fig. A.12. Disability-adjusted life years averted by 100 000 health workers, 100% target coverage, by regions. Modelled IPC interventions are enhancing hand hygiene (HH), increasing access to PPE (PPE only) and increasing access to PPE in combination with IPC training and education (PPE+). Dark blue represents years lived with disability; grey represents years of life lost. Error bars represent the 95% confidence intervals for each region.

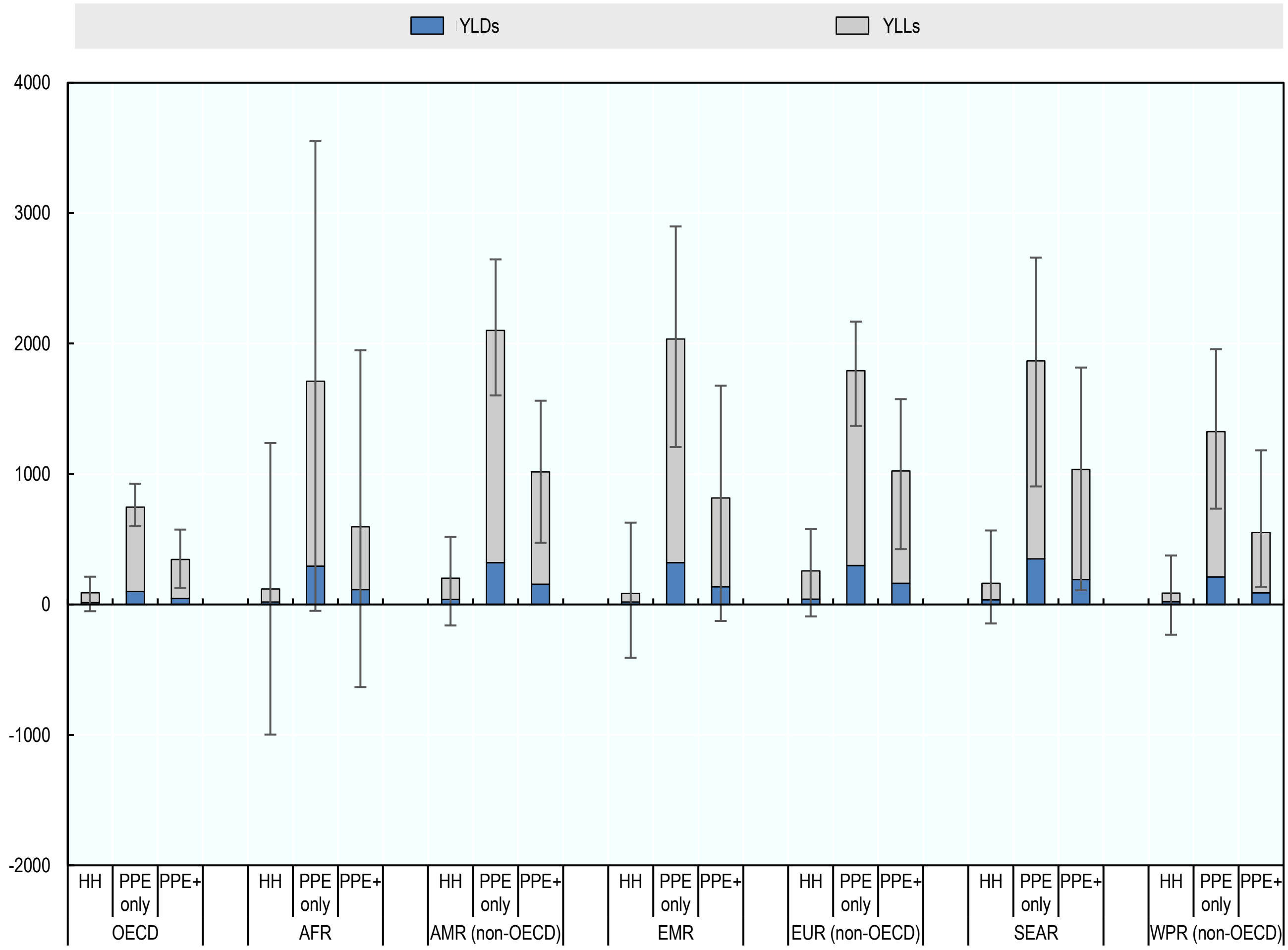

Fig. A.13. Costs and savings associated with scaling-up the modelled IPC interventions, 100% target coverage, by regions. Modelled IPC interventions are enhancing hand hygiene (HH), increasing access to PPE (PPE only) and increasing access to PPE in combination with IPC training and education (PPE+). Purple represents health expenditure; grey represents absenteeism; light blue represents premature mortality and dark blue represents total cost. Error bars represent the 95% confidence intervals showing the uncertainty associated with total savings and total

costs. Savings are expressed in positive US dollars values and costs are expressed in negative US dollar values.

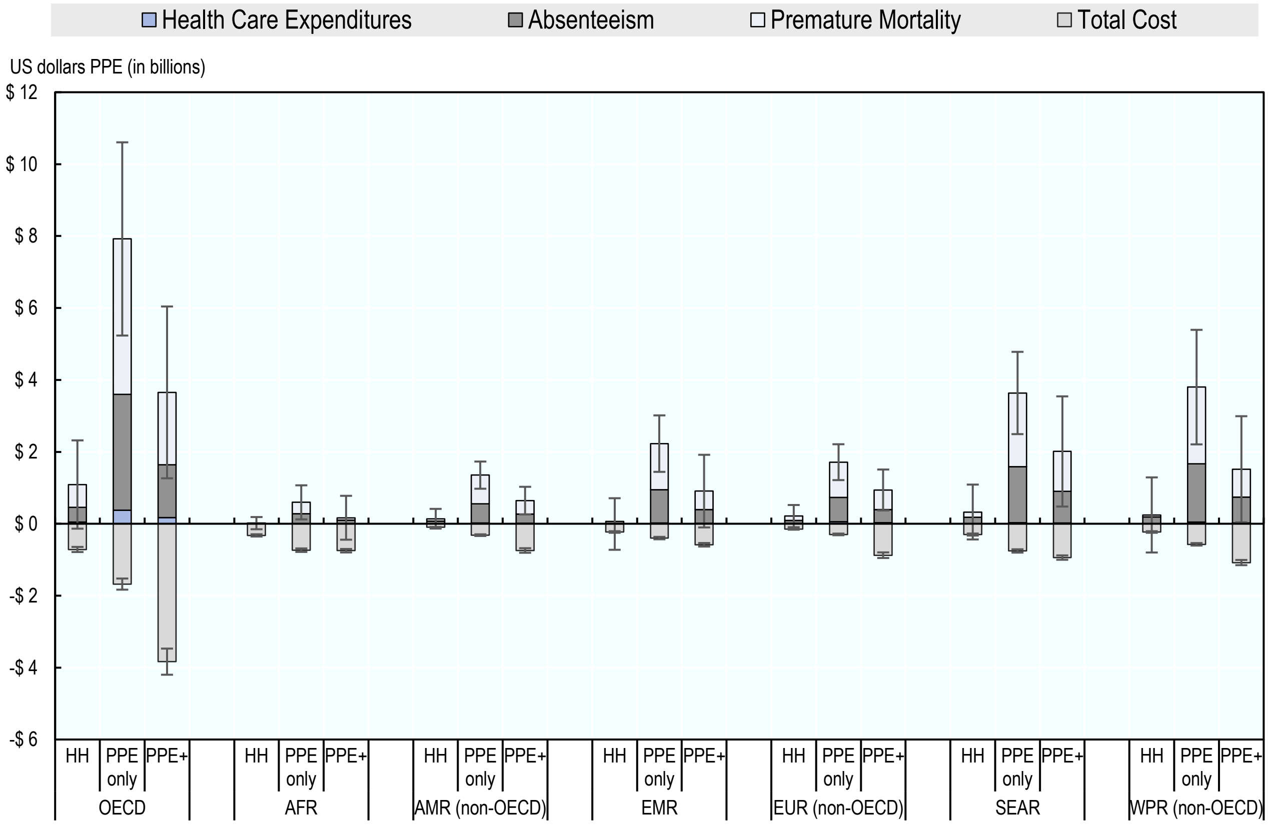

Probability of cost-effectiveness of the modelled interventions

The probability of cost-effectiveness of the modelled interventions by region are shown in Table A.10.

Table A.10. Probability of cost-effectiveness of three IPC interventions modelled in the study vs. a *business-as-usual* scenario.

**Intervention**	**WHO-OECD regions**						
HH	**OECD**	**AFR**	**AMR (non-OECD)**	**EMR**	**EUR (non-OECD)**	**SEAR**	**WPR (non-OECD)**
Cost-saving	0.39	0.37	0.49	0.15	0.48	0.42	0.49
Cost-effective (ICER ≤ CE threshold)	0.38	0.11	0.44	0.60	0.47	0.51	0.17
Not cost-effective (ICER > CE threshold)	0.23	0.52	0.07	0.26	0.06	0.07	0.34
PPE only	**OECD**	**AFR**	**AMR (non-OECD)**	**EMR**	**EUR (non-OECD)**	**SEAR**	**WPR (non-OECD)**
Cost-saving	0.65	0.11	0.86	0.72	1.00	0.98	0.86
Cost-effective (ICER < CE threshold)	0.34	0.22	0.14	0.28	0.00	0.02	0.13
Not cost-effective (ICER > CE threshold)	0.01	0.67	0.00	0.00	0.00	0.00	0.01
PPE +	**OECD**	**AFR**	**AMR (non-OECD)**	**EMR**	**EUR (non-OECD)**	**SEAR**	**WPR (non-OECD)**
Cost-saving	0.99	0.04	1.00	1.00	1.00	1.00	1.00
Cost-effective (ICER < CE threshold)	0.01	0.94	0.00	0.00	0.00	0.00	0.00
Not cost-effective (ICER > CE threshold)	0.00	0.03	0.00	0.00	0.00	0.00	0.00

HH; hand hygiene; PPE, personal protective equipment; OECD, Organisation for Economic Cooperation and Development; AFR, African region; AMR (non-OECD), non-OECD countries in the Americas region; EMR, Eastern Mediterranean region; non-OECD EUR, non-OECD countries in the European region; SEAR, South-East Asia region; non-OECD WPR, non-OECD countries in the Western Pacific region; ICER, incremental cost-effectiveness ratio, CE, cost effectiveness.

References

1. Rodda LB, Netland J, Shehata L, et al. Functional SARS-CoV-2-specific immune memory persists after mild COVID-19. Cell. 2021; 184:169-183.e17.
2. Gaebler C, Wang Z, Lorenzi JCC, et al. Evolution of antibody immunity to SARS-CoV-2. Nature. 2021; 591:639–44.
3. Walker PGT, Whittaker C, Watson OJ, et al. The impact of COVID-19 and strategies for mitigation and suppression in low- and middle-income countries. Science 2020; 369:413–22.
4. Salje H, Kiem CT, Lefrancq N, et al. Estimating the burden of SARS-CoV-2 in France. Science 2020; 369:208–11.
5. Jo MW, Go DS, Kim R, et al. The burden of disease due to COVID-19 in Korea using disability-adjusted life years. J Korean Med Sci 2020; 35.
6. Nurchis MC, Pascucci D, Sapienza M, et al. Impact of the burden of COVID-19 in Italy: results of disability-adjusted life years (DALYs) and productivity loss. Int J Environ Res Public Health 2020; 17:4233.
7. Sanjay G, Mohanty K, Dubey M, et al. Impact of COVID-19 attributable deaths on longevity, premature mortality and DALY: estimates of USA, Italy, Sweden and Germany. medRxiv, 2022 July 07. https://doi.org/10.1101/2020.07.06.20147009 (accessed Nov 9, 2022).
8. European Burden of Disease Network. Burden of disease of COVID-19 protocol for country studies. 2021. https://www.burden-eu.net/docs/covid19-bod-protocol.pdf (accessed Nov 9, 2022).
9. Haagsma JA, de Noordhout CM, Polinder S, et al. Assessing disability weights based on the responses of 30,660 people from four European countries. Popul Health Metr. 2015; 13:1–15.
10. Salomon JA, Haagsma JA, Davis A, et al. Disability weights for the Global Burden of Disease 2013 study. Lancet Glob Health. 2015;3:e712–23.
11. Office for National Statistics. Prevalence of ongoing symptoms following coronavirus (COVID-19) infection in the UK: 1 April 2021. 2021. https://www.ons.gov.uk/peoplepopulationandcommunity/healthandsocialcare/conditionsanddiseases/bulletins/prevalenceofongoingsymptomsfollowingcoronaviruscovid19infectionintheuk/1april2021 (accessed Nov 9, 2022).
12. Mattke S, Balakrishnan A, Bergamo G, Newberry SJ. A review of methods to measure health-related productivity loss. Am J Manag Care. 2007; 13.
13. OECD. Modelling work in public health: the OECD's SPHeP models.2021. https://www.oecd.org/health/modelling-work-in-public-health.htm (accessed Nov 9, 2022).
14. Lopez-Leon S, Wegman-Ostrosky T, et al. More than 50 long-term effects of COVID-19: a systematic review and meta-analysis. medRxiv Prepr Serv Heal Sci. 2021 Jan 30. https://pubmed.ncbi.nlm.nih.gov/33532785/ (accessed Nov 9, 2022).
15. Serje J, Bertram MY, Brindley C, Lauer JA. Global health worker salary estimates: an econometric analysis of global earnings data. Cost Eff Resour Alloc. 2018; 16:1–9.
16. McPeake J, Mikkelsen ME, Quasim T, et al. Return to employment after critical illness and its association with psychosocial outcomes: A systematic review and meta-analysis. Ann Am Thorac Soc. 2019; 16:1304–11.
17. Su H, Dreesmann NJ, Hough CL, Bridges E, Thompson HJ. Factors associated with employment outcome after critical illness: Systematic review, meta-analysis, and meta-regression. J Adv Nurs. 2021;77:653–63.
18. Quigley AL, Stone H, Nguyen PY, Chughtai AA, MacIntyre CR. Estimating the burden of COVID-

19 on the Australian healthcare workers and health system during the first six months of the pandemic. Int J Nurs Stud. 2021; 114..

19. Fenton L, Gribben C, Caldwell D, et al. Risk of hospital admission with covid-19 among teachers compared with healthcare workers and other adults of working age in Scotland, March 2020 to July 2021: population based case-control study. BMJ 2021; 374:2060.
20. WHO. National Health Workforce Accounts. Geneva: World Health Organization; 2021. https://apps.who.int/nhwaportal/ ((accessed Nov 9, 2022).
21. United Nations Population Division. World Population Prospects 2021. New York: United Nations 2021. https://population.un.org/wpp/ (accessed Nov 9, 2022).
22. World Bank. World Development Indicators Database. 2020. https://databank.worldbank.org (accessed Nov 9, 2022).
23. Honaker J, King G, Blackwell M. AMELIA II: A program for missing data. 2012. http://gking.harvard.edu/stats.shtml (accessed Nov 9, 2022).
24. WHO. The impact of COVID-19 on health and care workers: a closer look at deaths. (WHO/HWF/WorkingPaper/2021.1). Geneva: World Health Organization; 2021 https://apps.who.int/iris/handle/10665/345300 (accessed Nov 9, 2022).
25. Tomczyk S, Twyman A, de Kraker MEA, et al. The first WHO global survey on infection prevention and control in health-care facilities. Lancet Infect Dis. 2022;22:845–56.
26. WHO. Making choices in health : WHO guide to cost-effectiveness analysis. Tan-Torres Edejer T, et al (eds). Geneva: World Health Organization; 2003. https://apps.who.int/iris/handle/10665/42699 (accessed Nov 9, 2022).
27. Ran L, Chen X, Wang Y, Wu W, Zhang L, Tan X. Risk factors of healthcare workers with coronavirus disease 2019: a retrospective cohort study in a designated hospital of Wuhan in China. Clin Infect Dis, 2020; 71:2218–21.
28. Kassem AM, Talaat H, Shawky S, et al. SARS-CoV-2 infection among healthcare workers of a gastroenterological service in a tertiary care facility. Arab J Gastroenterol. 2020; 21:151–5.
29. Khalil MM, Alam MM, Arefin MK, et al. Role of personal protective measures in prevention of COVID-19 spread among physicians in Bangladesh: a multicenter cross-sectional comparative study. SN Compr Clin Med, 2020; 2:1733–9.
30. Jefferson T, Del Mar CB, Dooley L, et al. Physical interventions to interrupt or reduce the spread of respiratory viruses. Cochrane Database Syst Rev. 2011; 2020(4). https://www.cochranelibrary.com/cdsr/doi/10.1002/14651858.CD006207.pub4/full (accessed Nov 9, 2022).
31. Chou R, Dana T, Buckley DI, Selph S, Fu R, Totten AM. Update alert 10: Epidemiology of and risk factors for coronavirus infection in health care workers. Ann Intern Med. 2022; 175:W8–9.
32. Saunders-Hastings P, Crispo JAG, Sikora L, Krewski D. Effectiveness of personal protective measures in reducing pandemic influenza transmission: a systematic review and meta-analysis. Epidemics. 2017; 20:1–20.
33. White S, Thorseth AH, Dreibelbis R, Curtis V. The determinants of handwashing behaviour in domestic settings: an integrative systematic review. Int J Hyg Environ Health. 2020; 227:113512.
34. Burch J, Hammerschmidt J. What are the effects of multimodal campaigns to improve hand hygiene of healthcare workers? Cochrane Clin Answers 2019 Jan 29. https://www.cochranelibrary.com/cca/doi/10.1002/cca.2686/full (accessed Nov 9, 2022).
35. Houghton C, Meskell P, Delaney H, et al. Barriers and facilitators to healthcare workers' adherence with infection prevention and control (IPC) guidelines for respiratory infectious diseases: A rapid qualitative evidence synthesis. Cochrane Database Syst Rev. 2020; 4:1–55.

https://www.cochranelibrary.com/cdsr/doi/10.1002/14651858.CD013582/full (accessed Nov 9, 2022).

36. Mertz D, Dafoe N, Walter SD, Brazil K, Loeb M. Effect of a multifaceted intervention on adherence to hand hygiene among healthcare workers: a cluster-randomized trial. Infect Control Hosp Epidemiol. 2010; 31:1170–6.

37. Luangasanatip N, Hongsuwan M, Limmathurotsakul D, , et al. Comparative efficacy of interventions to promote hand hygiene in hospital: systematic review and network meta-analysis. BMJ 2015; 351.

38. Gould DJ, Moralejo D, Drey N, Chudleigh JH, Taljaard M. Interventions to improve hand hygiene compliance in patient care. Cochrane Database Syst Rev. 2017;2017(9). https://www.cochranelibrary.com/cdsr/doi/10.1002/14651858.CD005186.pub4/full (accessed Nov 9, 2022).

39. Price L, MacDonald J, Melone L, et al. Effectiveness of national and subnational infection prevention and control interventions in high-income and upper-middle-income countries: a systematic review. Lancet Infect Dis. 2018;18:e159–71.

40. WHO. Minimum requirements for infection prevention and control programmes. Geneva: World Health Organization; 2019. https://www.who.int/publications/i/item/9789241516945 (accessed Nov 9, 2022).

41. WHO. Hand hygiene in outpatient and home-based care and long-term care facilities: a guide to the application of the WHO multimodal hand hygiene improvement strategy and the “My Five Moments For Hand Hygiene” approach. Geneva: World Health Organization; 2012. https://apps.who.int/iris/handle/10665/78060 (accessed Nov 9, 2022).

42. Moore LD, Robbins G, Quinn J, Arbogast JW. The impact of COVID-19 pandemic on hand hygiene performance in hospitals. Am J Infect Control. 2021; 49:30–3.

43. National Academies of Sciences, Engineering and Medicine. Rapid expert consultation on understanding causes of health care worker deaths due to the COVID-19 pandemic (December 10, 2020).Washington, DC: The National Academies Press. 2020. https://doi.org/10.17226/26018 (accessed Nov 9, 2022).

44. Chu DK, Akl EA, Duda S, et al. Physical distancing, face masks, and eye protection to prevent person-to-person transmission of SARS-CoV-2 and COVID-19: a systematic review and meta-analysis. Lancet. 2020]; 395:1973–87.

45. Richterman A, Meyerowitz EA, Cevik M. Hospital-Acquired SARS-CoV-2 Infection: Lessons for Public Health. JAMA. 2020; 324:2155–6.

46. Verbeek JH, Rajamaki B, Ijaz S, et al. Personal protective equipment for preventing highly infectious diseases due to exposure to contaminated body fluids in healthcare staff. Cochrane Database Syst Rev 2020 (4). https://www.cochranelibrary.com/cdsr/doi/10.1002/14651858.CD011621.pub4/full (accessed Nov 9, 2022).

47. United States Centers for Disease Control and Prevention. Infection control guidance for healthcare professionals about coronavirus (COVID-19). Atlanta, GA: CDC; 2020. https://www.cdc.gov/coronavirus/2019-ncov/hcp/infection-control.html (accessed Nov 9, 2022).

48. WHO. Technical specifications of personal protective equipment for COVID-19. Geneva: World Health Organization; 2020. https://www.who.int/teams/health-product-and-policy-standards/access-to-assistive-technology-medical-devices/medical-devices/ppe/ppe-covid (accessed Nov 9, 2022).

49. Fusco FM, Pisaturo M, Iodice V, et al. COVID-19 among healthcare workers in a specialist infectious diseases setting in Naples, Southern Italy: results of a cross-sectional surveillance study. J Hosp Infect. 2020; 105:596–600.

50. Zhou F, Li J, Lu M, et al. Tracing asymptomatic SARS-CoV-2 carriers among 3674 hospital staff:a cross-sectional survey. EClinicalMedicine. 2020;26: 100510.
51. Wang J, Liu F, Tan JBX, Harbarth S, Pittet D, Zingg W. Implementation of infection prevention and control in acute care hospitals in Mainland China - a systematic review. Antimicrob Resist Infect Control. 2019; 8:1–16.
52. Storr J, Twyman A, Zingg W, et al. Core components for effective infection prevention and control programmes: New WHO evidence-based recommendations. Antimicrob Resist Infect Control. 2017; 6:1–18.
53. Gerolemou L, Fidellaga A, Rose K, et al. Simulation-based training for nurses in sterile techniques during central vein catheterization. Am J Crit Care. 2014; 23:40–8.
54. Allen GB, Miller V, Nicholas C, et al. A multitiered strategy of simulation training, kit consolidation, and electronic documentation is associated with a reduction in central line-associated bloodstream infections. Am J Infect Control. 2014; 42:643–8.
55. European Centre for Disease Prevention and Control. Infection prevention and control and preparedness for COVID-19 in healthcare settings - sixth update. Guidance. Stockholm: ECDC: 2021. https://www.ecdc.europa.eu/en/publications-data/infection-prevention-and-control-and-preparedness-covid-19-healthcare-settings (accessed Nov 9, 2022).
56. Barsuk JH, Cohen ER, Potts S, et al. Dissemination of a simulation-based mastery learning intervention reduces central line-associated bloodstream infections. BMJ Qual Saf. 2014; 23:749–56.
57. OECD. Stemming the superbug tide: just a few dollars more. Paris: OECD; 2018. https://www.oecd-ilibrary.org/social-issues-migration-health/stemming-the-superbug-tide_9789264307599-en (accessed Nov 9, 2022).
58. WHO. WHO COVID-19 Essential Supplies Forecasting Tool (COVID-ESFT) v4.1. COVID-19: Essential resource planning. Geneva: World Health Organization; 2022. https://www.who.int/publications/i/item/WHO-2019-nCoV-Tools-Essential_forecasting-2022.1 (accessed Nov 9, 2022).
59. Risko N, Werner K, Offorjebe OA, Vecino-Ortiz AI, Wallis LA, Razzak J. Cost-effectiveness and return on investment of protecting health workers in low- and middle-income countries during the COVID-19 pandemic. PLoS One. 2020; 15:e0240503.
60. Bilinski A, Salomon JA, Giardina J, Ciaranello A, Fitzpatrick MC. Passing the test: a model-based analysis of safe school-reopening strategies. Ann Intern Med. 2021; 174:1090–100.
61. Korber B, Fischer WM, Gnanakaran S, et al. Tracking changes in SARS-CoV-2 spike: evidence that D614G increases infectivity of the COVID-19 virus. Cell. 2020; 182:812-827.e19.
62. Kirby T. New variant of SARS-CoV-2 in UK causes surge of COVID-19. Lancet Respir Med. 2021; 9:e20–1.
63. Toyoshima Y, Nemoto K, Matsumoto S, Nakamura Y, Kiyotani K. SARS-CoV-2 genomic variations associated with mortality rate of COVID-19. J Hum Genet, 2020; 65:1075–82.

2022 CHEERS Checklist

Topic	No.	Item	Location where item is reported
**Title**			
	1	Identify the study as an economic evaluation and specify the interventions being compared.	Title is listed in Page 1
**Abstract**			
	2	Provide a structured summary that highlights context, key methods, results, and alternative analyses.	Abstract is available in Page 2
**Introduction**			
**Background and objectives**	3	Give the context for the study, the study question, and its practical relevance for decision making in policy or practice.	Background is available in pages 8-9.
**Methods**			
**Health economic analysis plan**	4	Indicate whether a health economic analysis plan was developed and where available.	Analysis plan is available in pages 9-14 and in the Appendix
**Study population**	5	Describe characteristics of the study population (such as age range, demographics, socioeconomic, or clinical characteristics).	Study population is clarified in Page 8
**Setting and location**	6	Provide relevant contextual information that may influence findings.	Location is available in Page 8
**Comparators**	7	Describe the interventions or strategies being compared and why chosen.	Comparator is defined as business as usual scenario in Page 12.
**Perspective**	8	State the perspective(s) adopted by the study and why chosen.	Perspective is stated in Page 13.
**Time horizon**	9	State the time horizon for the study and why appropriate.	Time horizon is listed in Page 8

Topic	No.	Item	Location where item is reported
**Discount rate**	10	Report the discount rate(s) and reason chosen.	No discount rate used considering the time horizon.
**Selection of outcomes**	11	Describe what outcomes were used as the measure(s) of benefit(s) and harm(s).	Page 12. More details on study outcomes were provided in the appendix
**Measurement of outcomes**	12	Describe how outcomes used to capture benefit(s) and harm(s) were measured.	Appendix provides details on the measurement of outcome variables.
**Valuation of outcomes**	13	Describe the population and methods used to measure and value outcomes.	Appendix provides details on the valuation of outcomes.
**Measurement and valuation of resources and costs**	14	Describe how costs were valued.	Pages 12 and 13 describes how costs were calculated. Appendix provides further information.
**Currency, price date, and conversion**	15	Report the dates of the estimated resource quantities and unit costs, plus the currency and year of conversion.	Page 13 describes the currency and date.
**Rationale and description of model**	16	If modelling is used, describe in detail and why used. Report if the model is publicly available and where it can be accessed.	Pages 9-12 describes the rationale and description of the model
**Analytics and assumptions**	17	Describe any methods for analysing or statistically transforming data, any extrapolation methods, and approaches for validating any model used.	Pages 10-11 describes the key modelling assumptions and Tables 1 and 2 provides details on the key model parameters and interventions modelled.
**Characterising heterogeneity**	18	Describe any methods used for estimating how the results of the study vary for subgroups.	Page 9 describes the regions used in the analysis. Appendix provides details on how the regions were developed.
**Characterising distributional effects**	19	Describe how impacts are distributed across different individuals or adjustments made to reflect priority populations.	No subgroup analysis were done beyond the geographic regions.
**Characterising uncertainty**	20	Describe methods to characterise any sources of uncertainty in the analysis.	Pages 13-14 describes the methodology for propagating uncertainty and model validation.

Topic	No.	Item	Location where item is reported
**Approach to engagement with patients and others affected by the study**	21	Describe any approaches to engage patients or service recipients, the general public, communities, or stakeholders (such as clinicians or payers) in the design of the study.	No engagement with patients or service recipients were undertaken.
**Results**			
**Study parameters**	22	Report all analytic inputs (such as values, ranges, references) including uncertainty or distributional assumptions.	Tables 1 and 2 provides all of the study parameters
**Summary of main results**	23	Report the mean values for the main categories of costs and outcomes of interest and summarise them in the most appropriate overall measure.	Pages 14-17 provides the summary of main results.
**Effect of uncertainty**	24	Describe how uncertainty about analytic judgments, inputs, or projections affect findings. Report the effect of choice of discount rate and time horizon, if applicable.	Appendix provides a set of sensitivity analyses to examine various effects of uncertainty.
**Effect of engagement with patients and others affected by the study**	25	Report on any difference patient/service recipient, general public, community, or stakeholder involvement made to the approach or findings of the study	No differences by patient/service recipients etc. were provided in the study.
**Discussion**			
**Study findings, limitations, generalisability, and current knowledge**	26	Report key findings, limitations, ethical or equity considerations not captured, and how these could affect patients, policy, or practice.	Pages 18-23 provides an in-depth discussion on key findings, limitations etc.
**Other relevant information**			
**Source of funding**	27	Describe how the study was funded and any role of the funder in the identification, design, conduct, and reporting of the analysis	Page 14 describes the source of funding.
**Conflicts of interest**	28	Report authors conflicts of interest according to journal or International Committee of Medical Journal Editors requirements.	Authors reported no conflict of interest.

From: Husereau D, Drummond M, Augustovski F, et al. Consolidated Health Economic Evaluation Reporting Standards 2022 (CHEERS 2022) Explanation and Elaboration: A Report of the ISPOR CHEERS II Good Practices Task Force. Value Health 2022;25.
doi:10.1016/j.jval.2021.10.008
